# Long-Term Outcomes of an International Cooperative Study of Intraoperative Radiotherapy Upfront Boost With Low Energy X-Rays in Breast Cancer

**DOI:** 10.3389/fonc.2022.850351

**Published:** 2022-03-17

**Authors:** Gustavo R. Sarria, Maria L. Ramos, Amalia Palacios, Ruben Del Castillo, Felipe Castro, Angel Calvo, Jose M. Cotrina, Adela Heredia, Jose A. Galarreta, Paola Fuentes-Rivera, Alicia Avalos, David A. Martinez, Kevin Colqui, Gonzalo Ziegler, Leonard Christopher Schmeel, Luis V. Pinillos, Frederik Wenz, Frank A. Giordano, Gustavo J. Sarria, Elena Sperk

**Affiliations:** ^1^ Department of Radiation Oncology, University Hospital Bonn, University of Bonn, Bonn, Germany; ^2^ Department of Mastology, Instituto Nacional de Enfermedades Neoplasicas, Lima, Peru; ^3^ Department of Radiation Oncology, University Hospital Reina Sofia, Cordoba, Spain; ^4^ Department of Radiation Oncology, Oncosalud-Auna, Lima, Peru; ^5^ Department of Radiotherapy, Instituto Nacional de Enfermedades Neoplasicas, Lima, Peru; ^6^ University Hospital Freiburg, University of Freiburg, Freiburg, Germany; ^7^ Department of Radiation Oncology, Mannheim Cancer Center, University Medical Center Mannheim, Medical Faculty Mannheim, Heidelberg University, Mannheim, Germany

**Keywords:** breast cancer, boost, intraoperative radiotherapy, IORT, whole-breast radiotherapy

## Abstract

**Purpose:**

The purpose of this study was to assess the effectivity of upfront kilovoltage intraoperative radiotherapy (IORT) as a boost in high-risk early-stage breast cancer patients from an international pooled cohort.

**Materials/Methods:**

Patients from four centers in three different countries were retrospectively screened. Those with a minimum 1-year follow-up were included. Cumulative local (LR), regional (RR), and distant metastasis rates (DM) were analyzed. Additionally, the estimated overall survival (OS) was assessed. The Cox regression analysis was performed to identify failure predicting factors.

**Results:**

A total of 653 patients from centers in Peru, Spain, and Germany were included. The median follow-up was 55 (12–180) months, and age was 58 (27–86) years. Clinical tumor (T) staging was T1 65.85%, T2 30.17%, and T3 3.98%. Positive margins were found in 7.9% and *in-situ* component in 20.06%. The median IORT dose was 20 (6–20). The median time from IORT to EBRT was 74.5 (13-364) days. An overall 3.4% (n = 22) of patients developed local recurrence at some point during follow-up. The 12-, 60-, and 120-month cumulative LR were 0.3%, 2.3%, and 7.9%, respectively. After multivariate analysis, only age <50 remained to be a significant prognostic factor for local recurrence (HR 0.19, 95% CI 0.08–0.47; p < 0.05). The 10-year estimated OS was 81.2%.

**Conclusion:**

Upfront boost with IORT yields similar local control outcomes to those EBRT-based reports. Results from prospective trials, regarding toxicity, cosmesis, and effectivity are awaited to confirm these findings.

## Highlights

•Intraoperative radiotherapy as an upfront boost for breast cancer seems effective
•Ten-year local control rates are similar to those of external beam radiotherapy
•After IORT-boost, only age remains to be a significant recurrence prognostic factor

## Introduction

Less aggressive approaches have been explored globally during the past few decades for breast cancer (BC) treatment (1). Particularly, breast-conserving surgery (BCS) in sequence with whole-breast radiotherapy (WBRT) was able to demonstrate comparable survival outcomes to those earlier radical surgery interventions (2). In the subset of high-risk patients, using a dose-escalated boost targeting the surgical bed has also demonstrated substantial benefits in terms of local control (3, 4), considering that up to 60% of BC patients might have boost indication (5).

Different methods for boost application have been reported, all of them demonstrating outstanding clinical results. Frequently, for external-beam radiotherapy (EBRT), sequential prescriptions of 40–50 Gy in 15 to 25 fractions are employed, summed to a 10–16-Gy boost delivered in 5–8 additional fractions. These schedules correspond to single 1.8- to 2.67-Gy daily doses, yielding in total 4 to 7 treatment weeks (6). Recently published and presented results of simultaneous integrated boost (SIB) techniques have been proposed to shorten treatment times within 15–16 fractions with hypofractionation (HF) (7, 8). Nevertheless, when it comes to EBRT-based boost delivery, this still carries a relatively fair cosmetic toll, plus the uncertainties of postsurgical tumor bed localization, when unmarked (9).

Intraoperative assessment of the surgical cavity under direct view represents the best option to accurately target residual tumor cells. In this regard, interstitial placing of multicatheter for high-dose rate brachytherapy (HDR) is one of the oldest and most studied techniques, with numerous publications and guidelines supporting it (10). One of its potential advantages, in comparison to EBRT, is a remarkably improved patient-reported cosmesis, perhaps as a result of reduced skin exposure (11). Nonetheless, HDR usually requires prolonged hospitalization times in order to fully deliver the required treatment fractions.

Under this same rationale, intraoperative radiotherapy (IORT), first as an electron- and lately as a kilovoltage-based (low-energy X-ray) approach, has recently risen as an additional option for these patients (12, 13). With a growing body of evidence, for both accelerated partial breast irradiation (APBI) and boost modalities, this technique allows a prompt irradiation of the surgical bed as an upfront boost within the same surgical procedure (14, 15). In addition, novel literature has suggested a potential enhancement of immune response, which might positively influence disease control outcomes (16).

Herein we report the combined experiences of four centers from Peru, Spain, and Germany, regarding disease control and survival after IORT upfront boost in BC patients.

## Methods

### Patients and Procedures

Patients from four radiotherapy centers in Peru (2), Spain (1), and Germany (1), negative for metastasis at debut, who received IORT as intended or unintended boost during BCS, between 2002 and 2019, and with a minimum 1-year follow-up time, were screened retrospectively and pooled for combined analysis. Inclusion criteria included age >18 years, unifocal invasive carcinoma (with or without *in-situ* components), and high-risk features requiring boost application (mentioned below). Immediately after tumor excision, kilovoltage (nominal 50 kV) (k)IORT was applied with the INTRABEAM (Carl Zeiss Meditec AG, Oberkochen, Germany) portable linear accelerator, delivered through a spherical applicator in all cases. Dose prescription was performed at the applicator surface, allowing approximately 30% isodose deposit after 1 cm (17). Generally, the prescribed dose was 20 Gy; however, lower doses could be applied if deemed appropriate by the practitioner (e.g., distance to skin < 1 cm).

Acknowledging risk factors present at diagnosis [age ≤50, ≥T2, tumor grade (G) 3 or triple negative (TNBC) or HER2 subtypes] or post-pathology assessment, complementary WBRT was prescribed. Both EBRT normofractionation (1.8–2 Gy per fraction) and hypofractionation (2.5–2.67 Gy per fraction) were allowed in the analysis. The clinical staging was considered according to the AJCC TNM 7th edition criteria. Unintended boost was delivered to the latter subset of patients, if no compliance with the TARGIT-A criteria (besides age) was met (13).

Routine follow-up visits were performed according to local standards every 6 months for the first 2 years and annually afterward, including physical examination, mammography, and/or ultrasonography scans or magnetic resonance imaging (MRI), when required.

### Endpoints

The main endpoint is the cumulative local recurrence rate (LR), accounting for any ipsilateral in-breast tumor recurrence (IBTR), proven by imaging and/or biopsy. Secondary endpoints include cumulative regional (nodal) recurrence rates (RR), distant metastasis rates (DM), defined as recurrence at any non-breast or regional lymphatic site, and overall survival (OS). Median and estimated times were calculated from the surgery date until any event occurrence or censoring. The relationship between risk factors and events is explored, as well. No toxicity assessment was considered for this study, due to heterogeneity in reporting among the participating centers.

### Statistical Analysis

Median times and ranges are displayed for descriptive purposes when required. LR are reported in relative values. The 1-, 5-, and 10-year cumulative LR, RR, DM and estimated OS were analyzed through the Kaplan–Meier method, and differences were assessed according to the Gehan, Traone-Ware, or log-rank tests, depending on the onset time point. The univariate and multivariate Cox regression models were employed to evaluate factors associated with the endpoints and expressed in hazard ratios (HR). The proportional hazard assumption was assessed, and a stratified analysis was performed for those variables not meeting the proportionality assumption. A p < 0.05 value was assumed to define statistically significant differences.

The analysis was performed with R Core Team (2021). R: A language and environment for statistical computing. R Foundation for Statistical Computing, Vienna, Austria. URL https://www.R-project.org/.

### Ethics Statement

The study was submitted individually to each local IRB prior to inclusion. After approval, anonymized data of patients who consented sharing from all four centers were collected. This investigation was performed according to the principles of the Declaration of Helsinki. No personal patient information is herein given.

## Results

### Cohort and Preoperative Features

A total of 653 patients were included in the final analysis with a median follow-up period of 55 (12–180) months. The median patient age was 58 (27–86) years, and left-sided BC were in 48.4% cases. Invasive ductal carcinoma/NST histology was diagnosed initially in 97.5% of patients, while definitive histology confirmed this diagnosis in 78.7% of the entire cohort. The initial clinical tumor (T) staging was T1 65.85%, T2 30.17%, and T3 3.98%, while nodal (N) staging was N0 79.48% and N1 20.52%. Tumor grading was G1 13.94%, G2 50.69%, G3 25.11%, and unknown in 8.12%. The luminal-like subtype proportion was 72.89%, HER2 16.08%, TNBC 10.57%, and unknown in 0.46%. Neoadjuvant chemotherapy was administered to 11.18% of patients.

### Surgical, Pathological, and IORT Features

The frozen section was required in 91.88% of patients, while in 23.12% of them, margin re-excision after IORT application was performed. An R1 margin situation was found in 7.9% of patients at the final pathology report. An *in-situ* associated component was observed in 20.06% and not reported in 8.27%. Margins from either invasive or *in-situ* lesions were ≥2 mm in 36.45% and <2 mm in 55.59%. The median (yp)tumor size was 15 (0–45) mm.

The median IORT dose was 20 (6–20) with 3.98% of patients receiving doses lower than 20 Gy. The median irradiation time was 26 (7.5–60) min, and the median applicator diameter was 4 (2–5) cm.

### Postsurgical and Adjuvant Treatment

Adjuvant endocrine therapy (ET) was received by 83.92% of patients. Additionally, 46.7% received adjuvant chemotherapy. EBRT was fully delivered to 99.54% of patients, of which 24.35% received hypofractionation. The median time elapsed since IORT until EBRT was 75 (13–364) days.

A summary of these features is observed in **Table 1**.

**Table 1:** Baseline patient characteristics.

	Absolute number of patients (n)	Proportion (%)		Absolute number of patients (n)	Proportion (%)
**Total**	653	100	**Total**	653	100
**Age, years**			**Confirmatory histology report**		
<50	159	24.35	IDC/NST	514	78.71
≥50	494	75.65	Other	139	21.29
**Side**			**Final margin report**		
Right	316	48.39	R0	600	91.88
Left	337	51.61	R1	52	7.96
**Initial histology**			NR	1	0.15
IDC/NST	637	97.55		**ypT**
Other	16	2.45	ypT0	28	4.29
**cT**			ypT1	435	66.62
cT1	430	65.85	ypT2	190	29.10
cT2	197	30.17		**ypN**
cT3	26	3.98	ypN0	501	76.72
**cN**			ypN1	152	23.28
cN0	519	79.48		**Grade**
cN1	134	20.52	G1	91	13.94
**Stage**			G2	331	50.69
IA	366	56.05	G3	164	25.11
IIA	206	31.55	NR	67	10.26
IIB	65	9.95	**Estrogen receptors**		
IIIC	16	2.45	Positive	549	84.07
**Neoadjuvant chemotherapy (any)**			Negative	100	15.31
Yes	73	11.18	NR	4	0.61
No	580	88.82	**Progesterone receptors**		
**Frozen section**			Positive	499	76.42
Yes	596	91.27	Negative	148	22.66
No	53	8.12	NR	6	0.92
NR	4	0.61		**HER2**
**Margin extension after IORT**			Positive	105	16.08
Yes	151	23.12	Negative	540	82.70
No	495	75.80	NR	8	1.23
NR	7	1.07		**Subtype**
**Median irradiation time**			TNBC	69	10.57
Minutes	26		Luminal A	395	60.49
Range	7.5–60		Luminal B	81	12.40
**Applicator diameter, cm**			HER2	105	16.08
≤3	100	15.31	NR	3	0.46
≥3.5	509	77.95	**Median tumor size (yp)**		
NR	44	6.74	Millimeters	15	
**IORT dose (Gy)**			Range	0 - 45	
20	627	96.02	***In-situ* component**		
< 20	26	3.98	Yes	131	20.06
**Endocrine therapy**			No	468	71.67
No	105	16.08	NR	54	8.27
Yes	548	83.92	**Margin from infiltrative/*in-situ* component**		
**Adjuvant chemotherapy**			≥ 2mm	238	36.45
No	348	53.29	<2 mm	162	24.81
Yes	305	46.71	NR	253	38.74
**Median/mean time from IORT to EBRT**					
Days	75/84			
Range/SD	13–364/ ± 110				
**EBRT fractionation**					
Hypofractionation	159	24.35			
Normofractionation	492	75.34			
NR	2	0.31			
**EBRT technique**					
3D conformal	337	51.61			
IMRT tangents	191	29.25			
VMAT	125	19.14			

IDC, infiltrative ductal carcinoma; NST, no special type; cT, clinical T stage; cN, clinical N stage; IORT, intraoperative radiotherapy; NR, not registered; SD, standard deviation; EBRT, external-beam radiotherapy; 3D, tridimensional; IMRT, intensity-modulated radiotherapy; VMAT, volumetric modulated radiotherapy; R0, negative microscopic margin; R1, positive microscopic margin; yp, post-neoadjuvant status; G, histologic grade; TNBC, triple-negative breast cancer; HER2, human epidermal growth factor receptor 2.

### Disease Control Outcomes

**Figure 1:**
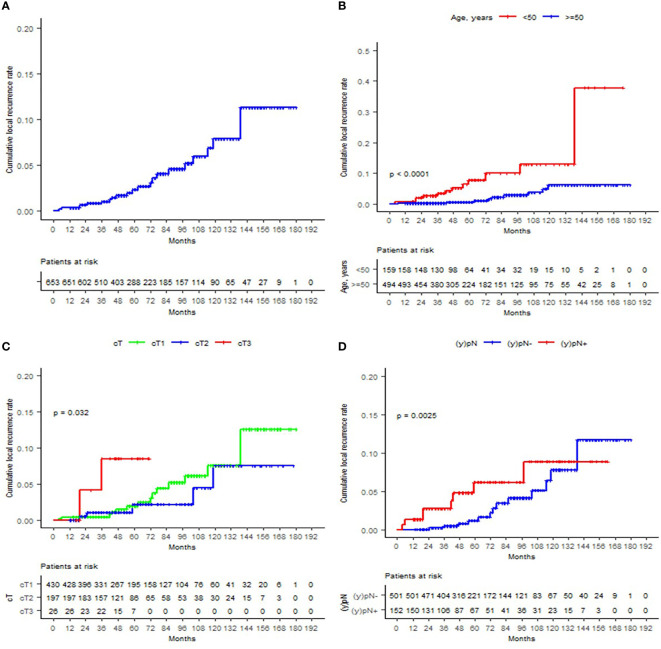
Cumulative local recurrence rates are displayed according to the Kaplan–Meier method for **(A)** the entire cohort, **(B)** age (<50 or ≥50), **(C)** T stage, and **(D)** ypN status.

As of the analysis, 62 patients (9.5%) died. 3.4% (n = 22) of patients developed local recurrence at some point of the entire follow-up period, with a median of 61 (4–139) months to IBTR. The 12-, 60-, and 120-month cumulative LR were 0.3%, 2.3%, and 7.9%, respectively. Significant LR differences were observed for age, T and clinical stages, ET, and post-neoadjuvant nodal status (ypN). In the univariate analysis, the significant predicting factors for IBTR were age, T stage, and ET. Only age ≥50 remained to be a significant predictor in the multivariate analysis (HR 0.19, 95% CI 0.08–0.47; p < 0.05). Margin re-excision after IORT application (p = 0.61), R1 situation (p = 0.54), resection margins <2 mm (p = 0.4), associated *in-situ* (p = 0.23), molecular subtype (p = 0.57), or WBRT hypofractionation (p = 0.34) was not related with increased recurrence. The Kaplan–Meier curves of the most relevant findings are displayed in **Figure 1**. These and further local control analyses are summarized in **Tables 2** and **3**.

**Table 2:** Local recurrence rates.

Local recurrence rates, %					
	N (events)	12 m	60 m	120 m	p
**All patients**	653 (22)	0.3	2.3	7.9	
**Age, years**					
<50	159 (13)	0.6	7.7	12.9	
≥50	494 (9)	0.2	0.5	6.1	**<0.0001**
**cT**					
cT1	430 (15)	0.5	1.9	7.6	
cT2	197 (5)	0.0	2.2	7.5	
cT3	26 (2)	0.0	8.5		**0.032**
**cN**					
cN0	519 (16)	0.2	1.5	8.0	
cN1	134 (6)	0.7	5.3	8.0	0.24
**Stage**					
I	372 (10)	0.0	0.9	6.5	
II	268 (11)	0.7	3.9	9.3	
III	13 (1)	0.0	8.3		**0.031**
**Margin extension after IORT?**					
Yes	151 (6)	0.0	1.4	13.2	
No	495 (16)	0.4	2.6	6.6	0.61
**Final margin status**					
R0	600 (20)	0.3	2.2	7.2	
R1	52 (2)	0.0	3.2	27.4	0.54
**Applicator diameter**					
≤3 cm	100 (4)	0.0	4.2	7.1	
≥3.5 cm	509 (13)	0.4	1.9	4.7	0.2
**Endocrine therapy**					
No	105 (6)	0.0	5.0	13.9	
Yes	548 (16)	0.4	1.9	7.1	**0.034**
**Adjuvant chemotherapy**					
No	348 (10)	0.0	0.4	7.4	
Yes	305 (12)	0.7	4.5	6.6	0.071
**Fractionation**					
Hypofractionation	159 (1)	0.0	1.8		
Normofractionation	492 (21)	0.4	2.5	8.1	0.34
**ypT**					
ypT0	28 (1)	0.0	9.1		
ypT1	435 (15)	0.5	1.7	7.4	
ypT2	190 (6)	0.0	2.9	8.3	0.89
**ypN**					
ypN0	501 (14)	0.0	1.1	7.8	
ypN1	152 (8)	1.3	6.2	8.8	**0.003**
**Grade**					
G1	91 (1)	0.0	0.0	4.2	
G2	331 (13)	0.6	2.2	8.5	
G3	164 (7)	0.0	4.8	10.1	0.33
***In situ* component**					
Yes	131 (4)	0.0	3.9	17.6	
No	468 (16)	0.4	2.3	6.9	0.23
**Margins from *in situ*/infiltrative component**					
≥2 mm	239 (8)	0.4	1.7	4.5	
<2 mm	162 (5)	0.0	3.8	8.6	0.4
**HER2**					
Positive	105 (3)	0.0	1.3	8.8	
Negative	540 (19)	0.4	2.5	7.7	0.75
**Molecular subtype**					
TNBC	68 (3)	0.0	4.7	4.7	
Luminal A	91 (3)	0.3	2.0	7.1	
Luminal B	80 (3)	1.2	2.9		
HER2	95 (3)	0.0	1.3	8.8	0.3

Variables related to LFS. Absolute number of patients and events, as well as the estimated 12-, 60-, and 120-month proportions and statistical significance are displayed.

IORT, intraoperative radiotherapy; cT, clinical T stage; cN, clinical N stage; R0, negative microscopic margin; R1, positive microscopic margin; yp, post-neoadjuvant status; G, histologic grade; TNBC, triple-negative breast cancer; HER2, human epidermal growth factor receptor 2. Bold text are significant p values.

**Table 3:** Cox regression analysis.

Cox regression analysis					
Variables	HR univariate	95% CI	p	HR multivariate	95% CI	p
**Age, years**						
<50	Ref.			Ref.		
≥50	0.18	0.07–0.41	**<0.05**	0.19	0.08–0.47	**<0.05**
**cT**						
cT1	Ref.			Ref.			
cT2	0.72	0.26–1.97	0.518	0.63	0.23–1.75	0.379
cT3	4.84	1.03–22.66	**0.045**	2.29	0.48–10.99	0.3
**Stage**						
I	Ref.					
II	1.57	0.67–3.71	0.3			
III	6.46	0.80–52.47	0.081			
**Endocrine therapy**						
No	Ref.			Ref.		
Yes	0.37	0.15–0.96	**0.041**	0.47	0.18–1.21	0.117
**ypN**						
ypN0	Ref.					
ypN1	2.05	0.85–4.89	0.108			

Uni- and multivariate analysis of risk factors related to local relapse.

HR, hazard ratio; CI, confidence interval; Ref, reference value; cT, clinical T stage; ypN, post-neoadjuvant nodal status. Bold text are significant p values.

The 12-, 60-, and 120-month cumulative RR were 0.5%, 1.2%, and 2.1%, respectively. Significant incidence differences were observed only for adjuvant chemotherapy use (p = 0.038). No uni- or multivariate analysis was performed due to the overall low number of events (n = 9).

A total of 6.74% (n = 44) patients developed distant progression during the follow-up period. The 12-, 60-, and 120-month cumulative DM were 0.6%, 6.9%, and 13.2%, respectively. Significant differences were observed for T staging (p = 0.032), N stage (p < 0.05), clinical stage (p = 0.001), adjuvant chemotherapy (p < 0.05), fractionation (favoring hypofractionation, p = 0.021), and ypN status (p < 0.05). The univariate analysis of risk factors determined that T3, N1, stages II and III, use of adjuvant chemotherapy, use of normofractionation, ypN1, and HER2 subtype were related to increased metastatic events. Only the HER2 subtype remained significant in the multivariate analysis (HER2-negative HR 0.47, 95% CI 0.25–0.89; p = 0.02).

**Figure 2:**
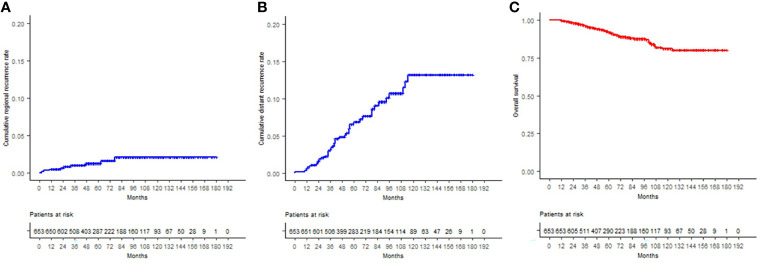
**(A)** Cumulative regional recurrence rates, **(B)** cumulative distant recurrence rates, and **(C)** overall survival curves are displayed for the entire cohort, according to the Kaplan–Meier method.

The estimated 12-, 60-, and 120-month OS was 99.8%, 91.9%, and 81.2%, respectively. Of the total deceased patients (62), 56.45% (n = 35) were related to disease progression, 40.32% to other causes, and no information was available for two cases (3.23%). Significant OS differences were found according to T, N, and clinical stages, ET, adjuvant chemotherapy, ypN status, tumor grade, and hormone receptor status. All the aforementioned were significant in the univariate analysis to predict death. In the multivariate analysis, T3 stage (HR 3.55, 95% CI 1.16–10.88; p = 0.027) and ypN1 (HR 3.22, 95% CI 1.04–9.94; p = 0.042) remained significant. RR, DM, and OS curves are shown in **Figure 2**.

Additional statistical reports are found in **Appendix A**.

## Discussion

Since the seminal studies by Veronesi et al., the paradigm of treating BC with modern conservative approaches was established as the new standard of care (1, 2). The role of WBRT plus boost, for patients with increased risk of IBRT, is currently a key piece within the arsenal to assure good oncologic outcomes (3). Upfront boosting techniques with IORT have been under investigation for over 50 years, with kIORT being the latest option on-scene. Historical data have already proven the advantages and long-term outcomes of electron-based IORT (IOERT) as a boost, leading to modern guidelines recommending its utilization for selected patients (18–20). Nowadays, different aspects sustain the rationale for breast IORT. From an immunological perspective, IORT acts on regulating the surgical cavity environment and pro-inflammatory cytokines, which might be triggered after surgical aggression (21, 22). An immediate downregulation of surgically induced tumor-growth factors could potentially yield improved disease-control outcomes, overcoming a temporal miss factor (23–25). Recent data have suggested an improvement in breast cancer mortality for those patients undergoing IORT; nevertheless, different confusing factors might be associated with these differences and further research is warranted to confirm this statement (13, 26). An interesting feature related to this cohort was the time elapsed between IORT and EBRT start. With a median 74.5 days (>10 weeks), it seems that results might not be impaired by this extended waiting time. It is important to remark that most of this waiting was due to sequencing between chemotherapy and EBRT. Another point lies on logistics and patients’ comfort. Shortening the treatment duration by at least 1 week has direct influence on treatment-related costs for both the patient and healthcare provider, while patient satisfaction increases due to time convenience and improved cosmesis (27–30). Furthermore, reducing the probability of geographical miss by applying radiotherapy directly into the surgical cavity under direct view could be hardly compared to postoperative surgical bed reconstruction, particularly when unmarked and mostly during the era of oncoplastic surgery (31). Despite these advantages, weighing the abovementioned with increased operatory times, longer anesthesia, and surgeon availability should be formally analyzed to define the actual cost-effectivity profile of this intervention.

Our group reports the largest cohort of patients receiving both WBRT and kIORT upfront boost, to date. Based on the previously mentioned, investigating this subject results to be compulsory in an era of shorter treatments and improvement in patients’ quality of life (QOL). Despite its retrospective nature, the cooperation established among centers from three different countries allows a real-world comparison of patients undergoing this treatment. Furthermore, the patients herein included were collected mostly in a prospective fashion, either withdrawn from clinical trials due to screening failures or collected in institutional registries. This feature allowed a quite homogeneous data collection and further interpretation. Notwithstanding, no secondary event assessment was considered due to differences in registration, which largely differed according to local standards. Regarding the latter, previous publications have highlighted a possible equivalence, if not superiority, of combined IORT-EBRT against pure EBRT approaches. An early report by Kraus-Tiefenbacher et al. described similar toxicity profiles, in comparison to historical EBRT data, for a prospectively collected cohort of 73 patients (32). Recently, various reports have reached consistent findings, with median follow-up periods of up to 78 months (14); moreover, data suggested improved cosmetic outcomes when compared to EBRT-boost trials. For example, data pooled from the EORTC 22881/10882 trial demonstrated after physician-based assessment good/excellent cosmetic results in 71% of patients, while kIORT and IOERT data yielded rates over 90% and 86%, respectively (32–35), similar to those obtained after HDR application (36). Additionally, complementary hypofractionated EBRT does not seem to impair cosmetic outcomes, as recently reported by Burgos-Burgos et al. (37)

This patient cohort had rather unfavorable prognostic factors as those described by prospective trials, including ~26% TNBC/HER2, ~34% ≥T2, and ~8% R1, among others. As the main purpose of boost application is increasing local tumor control, the central endpoint to assess in this trial was LR and LFS (6). With a median follow-up period of 55 months, the cumulative recurrence rate herein obtained of 3.4% resembles those reported by the EORTC trial, about 4.3% after 5 years, or the 2.8% reported by the START-B trial. In addition, the former reported a 10-year cumulative recurrence rate of 6.4% compared to 7.9% LR in our study (3, 38, 39). Although this comparison results interesting, no conclusion could be assumed due to methodological and sample size differences (2,661 vs. 653 patients). Nonetheless, interesting hypotheses could be pondered after these findings. Other experiences published along the last decade, assessing kIORT as a boost approach, have reached similar and encouraging outcomes with follow-up times between 28 and 78 months (14, 40–43). In spite of recurrence risks, classical factors, such as patient age <50, higher T and clinical stages, ET, and ypN1 outcome, were related to increased failure rates. In the multivariate analysis, only age was a significant predictor for IBTR. We found with great surprise that other variables, which might be as well associated with failure, such as re-excision after IORT, R1, and *in-situ* component and its margin, had no relationship with increased recurrence rates. Special attention should be given to re-excision and R1 situations. The former is a relatively common issue at centers practicing IORT, as this has been reportedly performed after IORT application and receiving frozen-section notice. In our study, 151 patients recorded this feature. This is probably due to the surgeon’s preference for reducing surgical time and self-confidence in obtaining an upfront negative margin after primary excision. Depending on the extent of resection, this could potentially impair control outcomes. However, these re-excision interventions are usually focused on the compromised surgical coordinates, while the rest of the cavity remains unmodified after IORT. It is worth mentioning that approximately 30% of the applied dose reaches a 1-cm depth (17), which according to the previously pondered might still be active on the re-excised area. Regarding the latter, R1 has classically pointed the need of dose escalation to achieve comparable control rates to those R0 margins (44). The boost doses applied to these patients, mostly 20 Gy, should suffice to cover the entire clinical area of interest, considering the surface delivery and penetration depth of kIORT. Another item to consider, although not clinically proven, is the relative biological effectiveness (RBE), which might increase the effectivity of IORT at a cellular interaction level. Typically, an average 1.3 factor is employed in addition to 2-Gy per fraction equivalent dose (EQD_2_) calculations to obtain equivalent doses to those of EBRT (45). We must remark that high dose-per-fraction EQD_2_ calculations carry flaws and cautious interpretation of these data should be done. Despite these physical uncertainties, the growing body of clinical information demonstrates the value of the IORT approach. A further feature was fractionation. We observed no differences between hypo- and normofractionation, in terms of local control. Modern approaches including SIB are currently being investigated, aiming at shortening treatment times to the nowadays 3-week standard timespan for low-risk BC patients (7, 8). Future investigations will be required to assess whether upfront boosting with IORT might confer clinically relevant immune enhancement compared to EBRT.

Further variables related to LR, DM, and OS were found accordingly to those published by larger series (46). It drew our attention the large differences in survival among fractionation schemes, favoring hypofractionation. We must note the significant disparity in follow-up times between them, as hypofractionation was initiated later in time. This variability resulted in 19/159 and 264/492 assessable patients after 5 years, respectively. Therefore, a relevant clinical meaning should be disregarded.

Certainly, this investigation burdens a number of limitations. Its retrospective nature and inherent shortcomings are to be mentioned. No toxicity or cosmesis assessment was performed due to the heterogeneity of data collection and classification. Nonetheless, as previously mentioned, different groups have already reported these features. Patient-reported outcomes, added to physician objective assessment, are required to understand the actual toxicity extent and cosmetic implications of both kIORT and IOERT (47). Cancer control results should be interpreted according to the limitations of a retrospective assessment, as under no circumstances should it be assumed that IORT would overcome well-established recurrence risk features. Moreover, these indirect inter-study comparisons against seminal series should be carefully interpreted and taken only for referential purposes. The rather small number of events did not allow a subgroup analysis to adequately identify recurrence-related variables. Despite these drawbacks, major strengths of our study lie on the number of included patients, as well as their multicenter and international character. Careful patient selection and timing between IORT and EBRT start, among others, are important elements to be considered for avoiding undesired toxicity (5, 48). These real-world data, although encouraging, should be assumed preliminary and hypothesis generating, while results from ongoing trials are expected (NCT01343459, NCT01792726).

## Conclusion

Upfront boost with IORT might yield similar local control outcomes to those reported by historical trials for high-risk early BC patients. Results from prospective trials, regarding toxicity, cosmesis, and effectivity, are awaited to confirm these findings.

## Data Availability Statement

The raw data supporting the conclusions of this article will be made available by the authors, without undue reservation.

## Author Contributions

GS: study conceptualization, data collection, curation, statistical analysis, manuscript drafting and editing. MR: study conceptualization and data collection. AP: data collection and curation. RC: data collection and curation. FC: data collection. AC: data collection. JC: study design and site supervision. AH: site supervision. JG: study design and site supervision. PF-R: study design and data collection. AA: study design and data collection. DM: data collection and site supervision. KC: data curation. GZ: site supervision. LS: manuscript review and editing. LP: manuscript review and site supervision. FW: study conceptualization, manuscript review, and editing. FG: manuscript review and editing. GS: study conceptualization and supervision. ES: study conceptualization, data collection, manuscript review, editing, and supervision. All authors contributed to the article and approved the submitted version.

## Conflict of Interest

GS: personal fees and travel costs from Carl Zeiss Meditec AG, personal fees from Roche Pharma AG, personal fees from MedWave Clinical Trials, travel costs from Guerbet SA, not related to this work. FW: reports personal fees from Celgene GmbH, fees Roche Pharma AG, fees Eli Lilly and Company, fees from Ipsen Pharma GmbH and grants and other from Carl Zeiss Meditec AG and Elekta AB, patent by Carl Zeiss Meditec AG, not related to this work, outside the submitted work. FG: grants and personal fees from Carl Zeiss Meditec AG, personal fees from Roche Pharma AG, grants and personal fees from Elekta AB, grants and personal fees from NOXXON Pharma AG, grants and personal fees from Bristol-Myers Squibb, grants and personal fees from MSD Sharp and Dome GmbH, grants and personal fees from AstraZeneca GmbH, non-financial support from ONCARE GmbH, non-financial support from OPASCA GmbH, outside the submitted work. GS: personal fees and travel costs from Carl Zeiss Meditec AG, not related to this work. ES: grants from the Ministry for Science and Arts and others from Carl Zeiss Meditec, outside of the submitted work.

The remaining authors declare that the research was conducted in the absence of any commercial or financial relationships that could be construed as a potential conflict of interest.

## Publisher’s Note

All claims expressed in this article are solely those of the authors and do not necessarily represent those of their affiliated organizations, or those of the publisher, the editors and the reviewers. Any product that may be evaluated in this article, or claim that may be made by its manufacturer, is not guaranteed or endorsed by the publisher.
