# Supplementary material for: Long-Term Outcomes of an International Cooperative Study of Intraoperative Radiotherapy Upfront Boost With Low Energy X-Rays in Breast Cancer
Source: Front Oncol. 2022 Mar 17;12:850351. doi: 10.3389/fonc.2022.850351 (PMC8968081; doi:10.3389/fonc.2022.850351)
Supplement: Supplementary file 1 [file DataSheet_1.pdf]

Appendix A

Table 1. Overall survival by patient's characteristics.

	Overall survival, %				
	N (events)	12m	60m	120m	p
**All patients**	653 (62)	99.8	91.9	81.2	
**Age, years**					
<50	159 (17)	100.0	88.2	77.7	
≥50	494 (45)	99.8	93.1	82.4	0.39
**cT**					
cT1	430 (36)	100.0	93.7	81.6	
cT2	197 (21)	99.5	90.3	82.0	
cT3	26 (5)	100.0	74.3		**0.014**
**cN**					
cN0	519 (39)	100.0	94.0	83.1	
cN1	134 (23)	99.3	84.3	74.4	**0.003**
**Stage**					
I	372 (26)	100.0	95.5	83.7	
II	268 (32)	99.6	88.0	79.1	
III	13 (4)	100.0	74.0		**<0.05**
**Frozen section before IORT?**					
Yes	596 (59)	99.8	92.2	81.4	
No	53 (2)	100.0	95.0		0.61
**Was margin extension performed after IORT?**					
Yes	151 (17)	100.0	88.7	82.7	
No	495 (45)	99.8	93.0	81.0	0.22
**Final paraffin "R" situation**					
R0	600 (56)	99.8	92.6	81.8	
R1	52 (6)	100.0	81.5	74.7	0.13
**Applicator diameter, cm**					

≤3	100 (9)	100.0	91.5	84.7	
≥3.5	509 (45)	99.8	92.1	81.3	0.53
**Hormone therapy**					
No	105 (14)	100.0	85.5	72.2	
Yes	548 (48)	99.8	93.0	82.6	**0.029**
**Adjuvant chemotherapy**					
No	348 (28)	100.0	94.2	85.7	
Yes	305 (34)	99.7	89.1	72.0	**0.018**
**Fractionation**					
Hypofractionation (>2Gy/Fx)	159 (3)	100.0	93.6		
Normofractionation (1.8-2Gy/Fx)	492 (59)	99.8	91.2	80.5	0.058
**(y)pT**					
(y)pT0	28 (3)	100.0	90.1		
(y)pT1	435 (39)	100.0	92.6	81.0	
(y)pT2	190 (20)	99.5	90.6	82.6	0.56
**(y)pN**					
(y)pN(-)	501 (36)	100.0	94.6	84.0	
(y)pN(+)	152 (26)	99.3	83.2	72.3	**<0.05**
**Grade**					
G1	91 (2)	100.0	100.0	91.7	
G2	331 (30)	99.7	92.5	84.3	
G3	164 (19)	100.0	88.3	77.1	**0.036**
**Estrogen receptors**					
Positive	549 (48)	99.8	92.9	82.3	
Negative	100 (14)	100.0	86.0	74.0	**0.029**
**Progesterone receptors**					
Positive	499 (43)	99.8	93.2	82.1	
Negative	148 (19)	100.0	87.2	77.7	**0.016**
**HER2**					
Positive	105 (13)	100.0	89.8	77.6	
Negative	540 (49)	99.8	92.3	81.7	0.25

**Subtype**					
Triple negative	68 (9)	100.0	86.7	72.0	
Luminal A	91 (4)	100.0	83.4		
Luminal B	80 (5)	100.0	93.5		
HER2,Luminal/HER2	95 (10)	100.0	91.4	79.0	0.53

Figure 1. Overall survival.

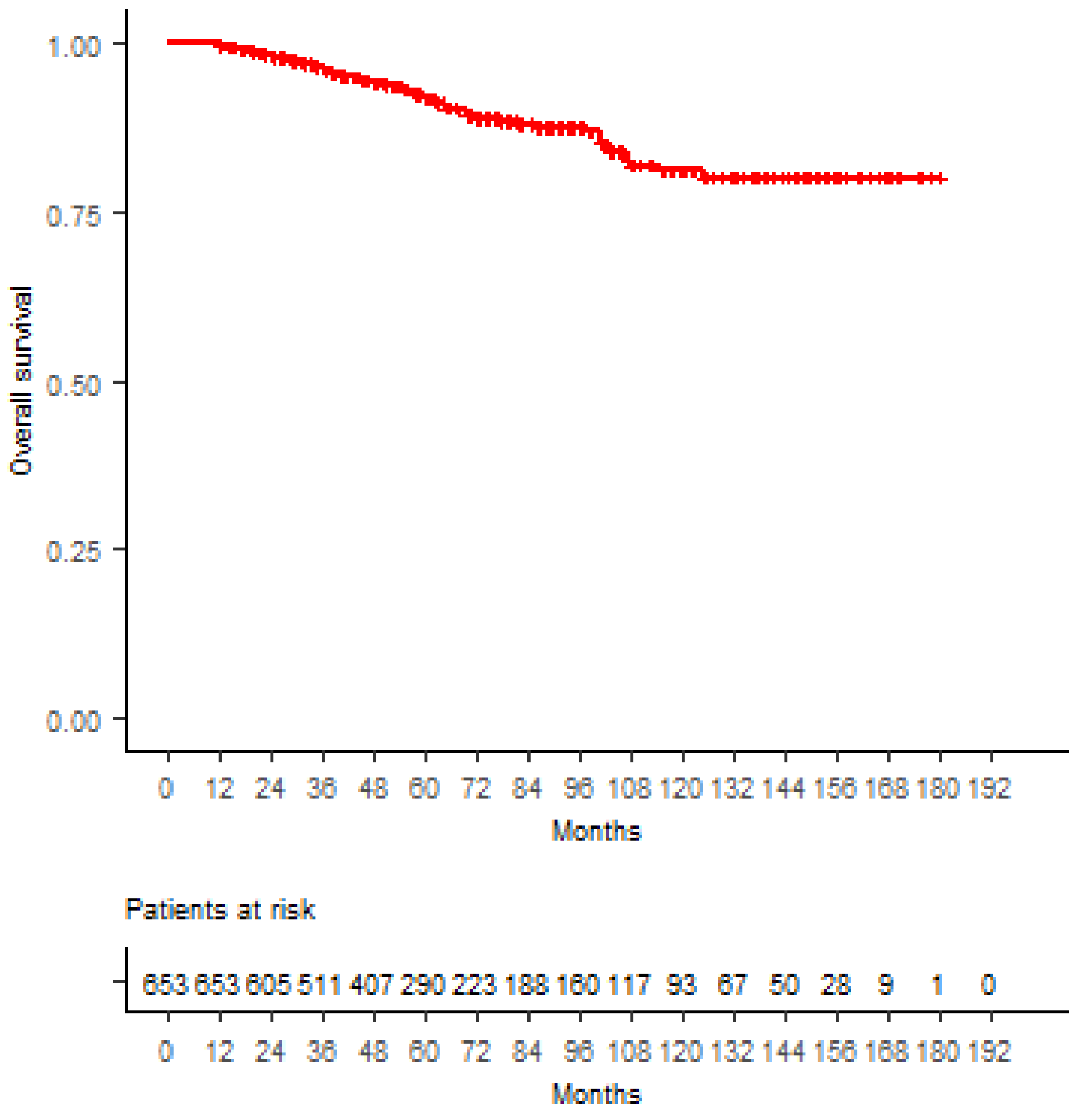

Figure 2. Overall survival according to cT.

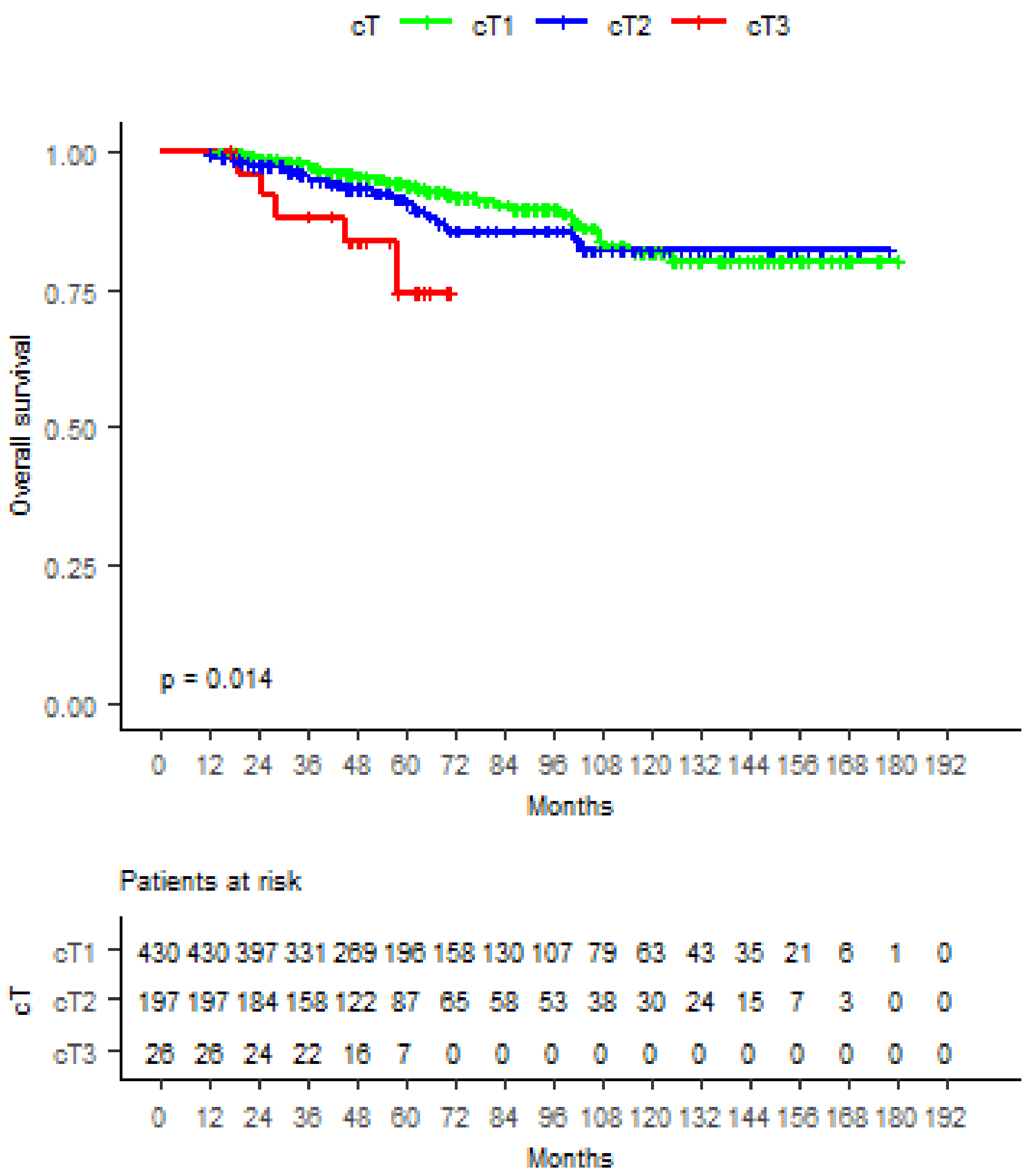

Figure 3. Overall survival according to cN.

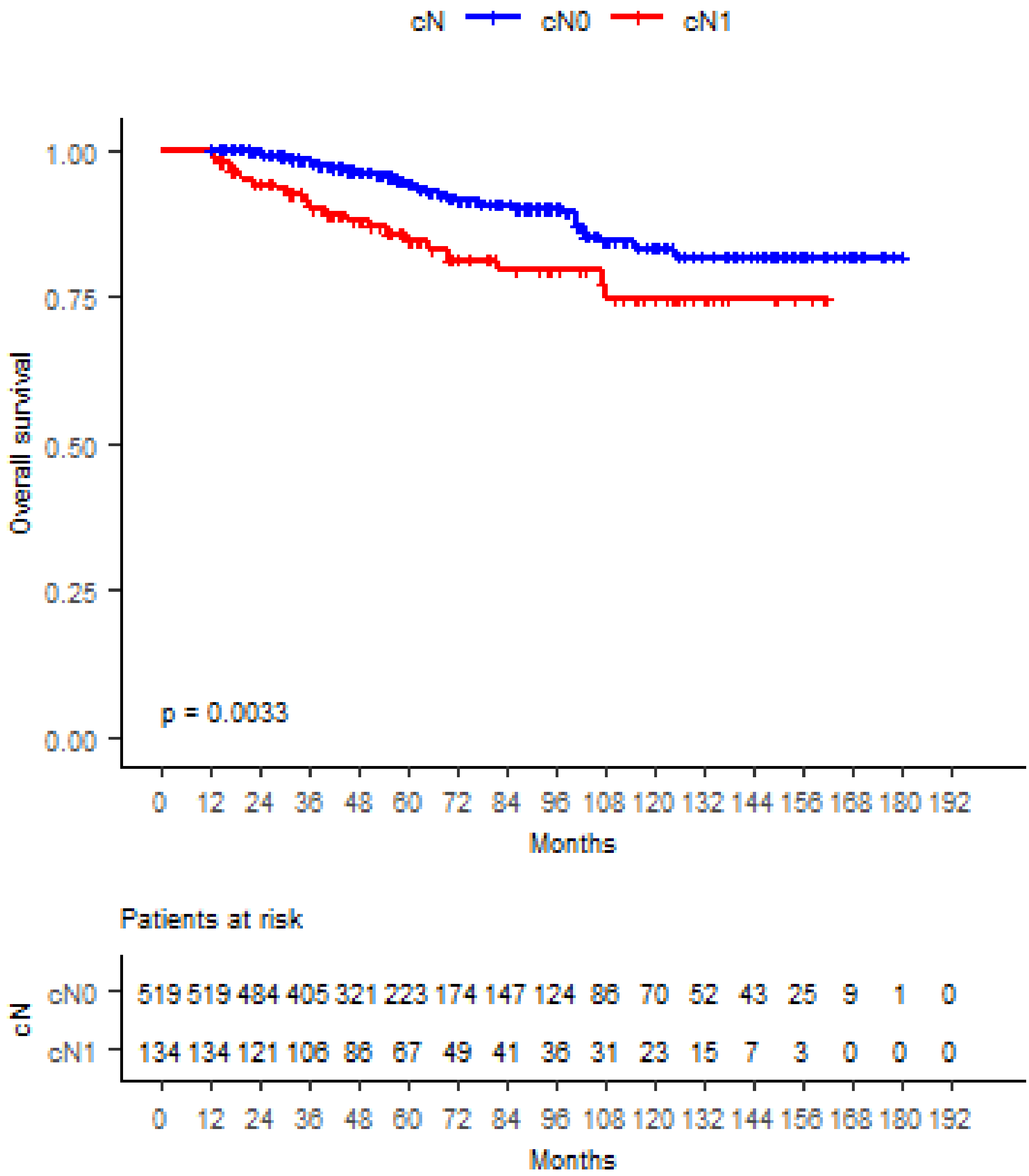

Figure 4. Overall survival according to stage.

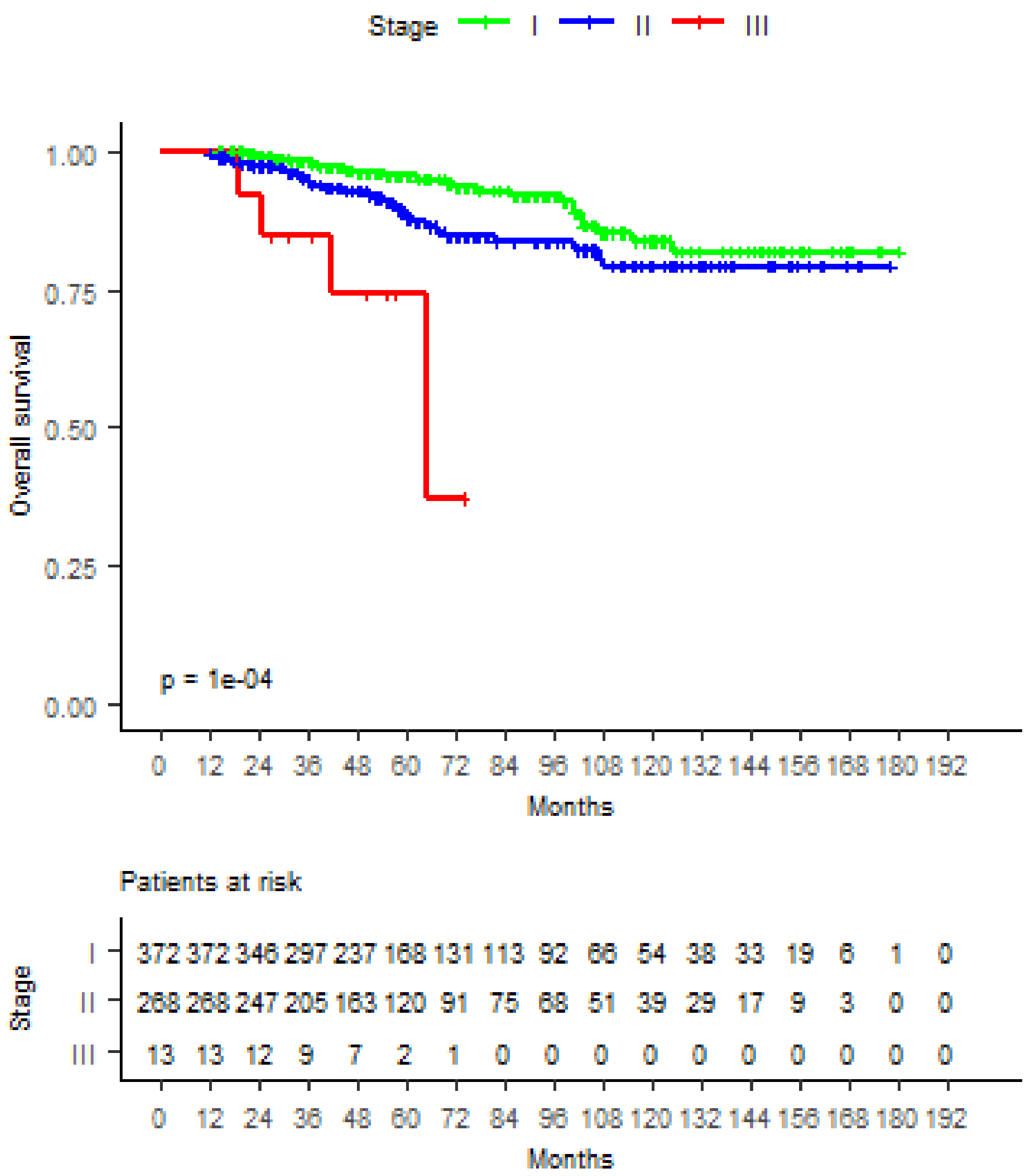

Figure 5. Overall survival with and without hormone therapy.

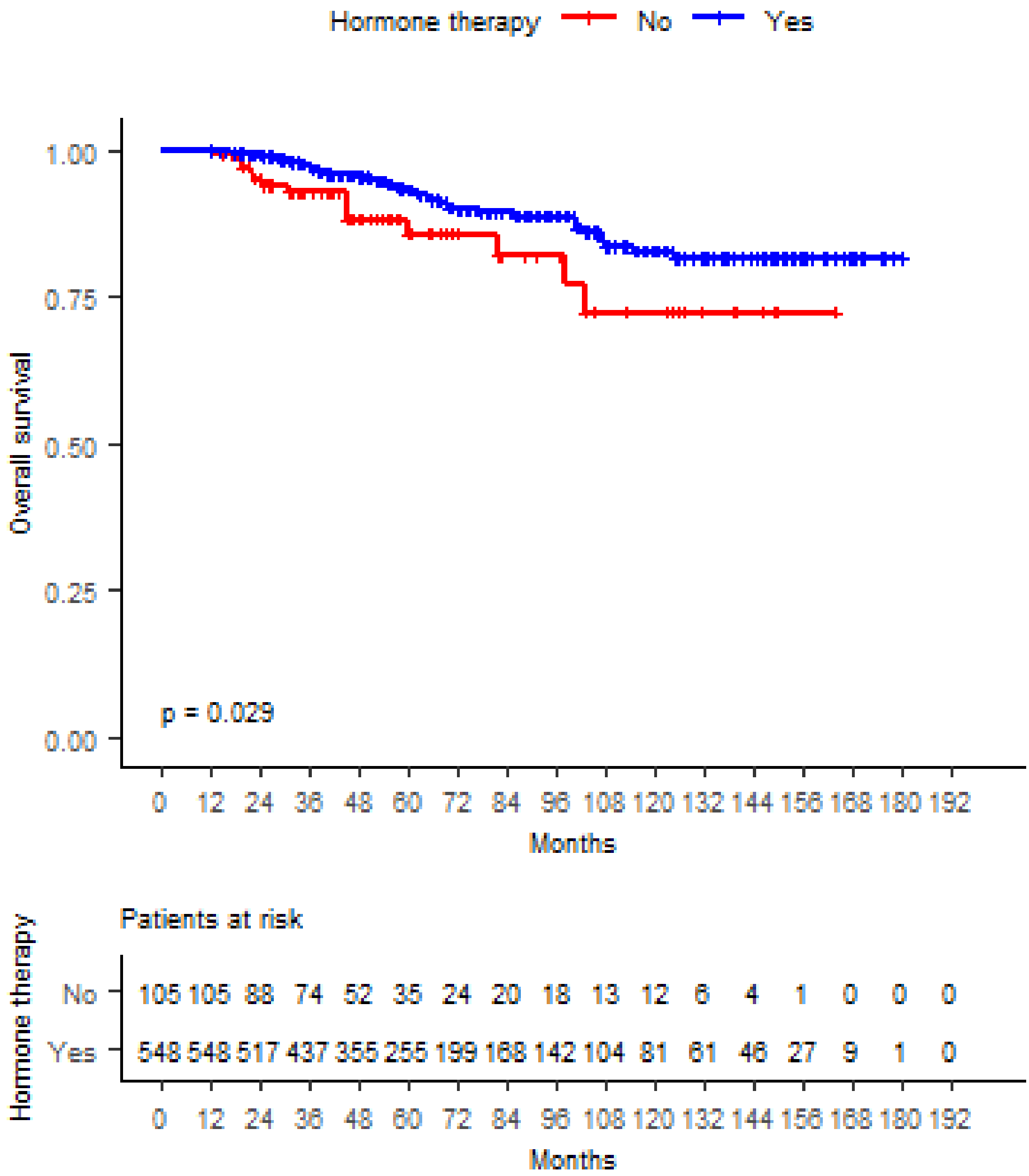

Figure 6. Overall survival with and without adjuvant chemotherapy.

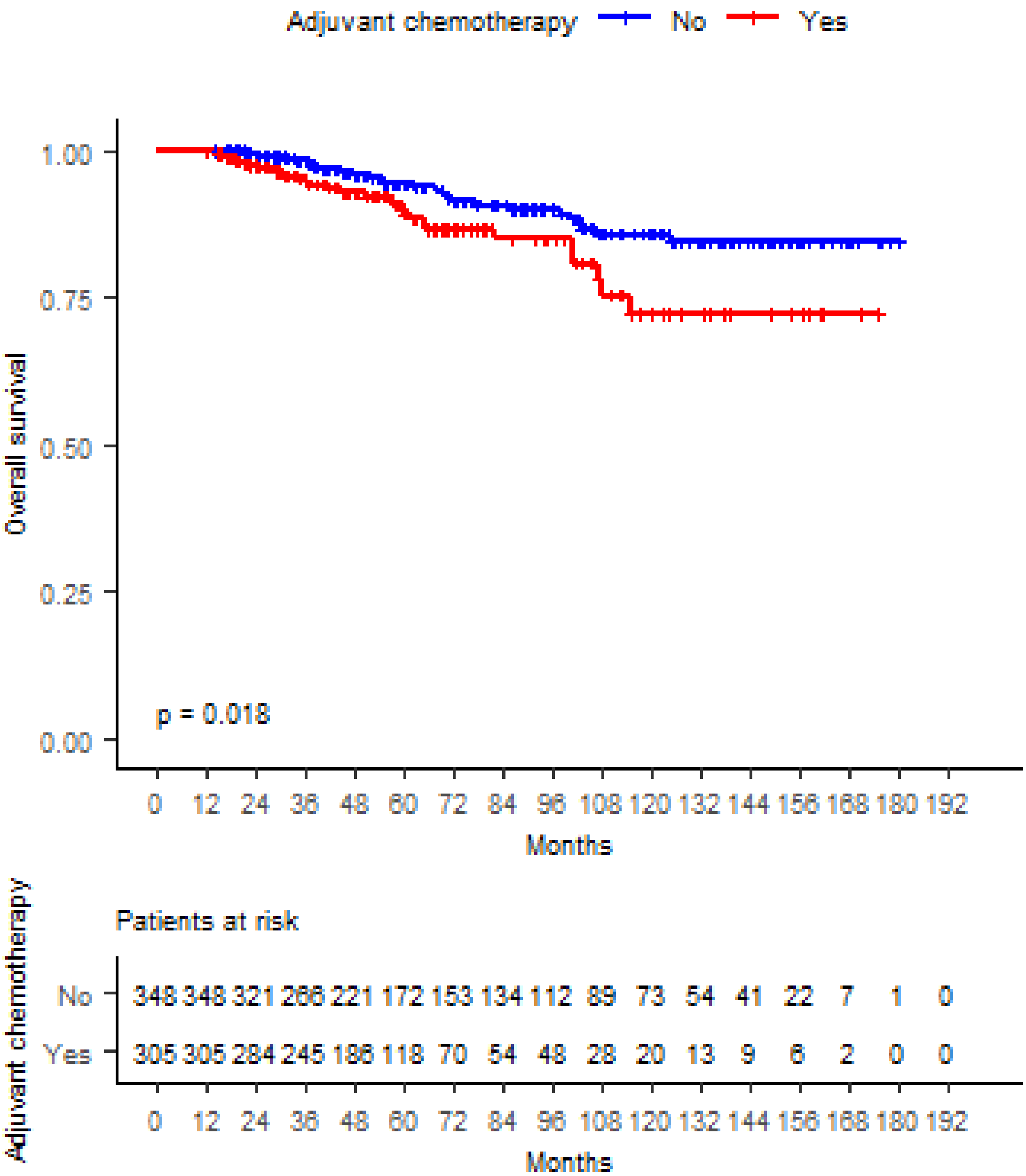

Figure 7. Overall survival according to pN.

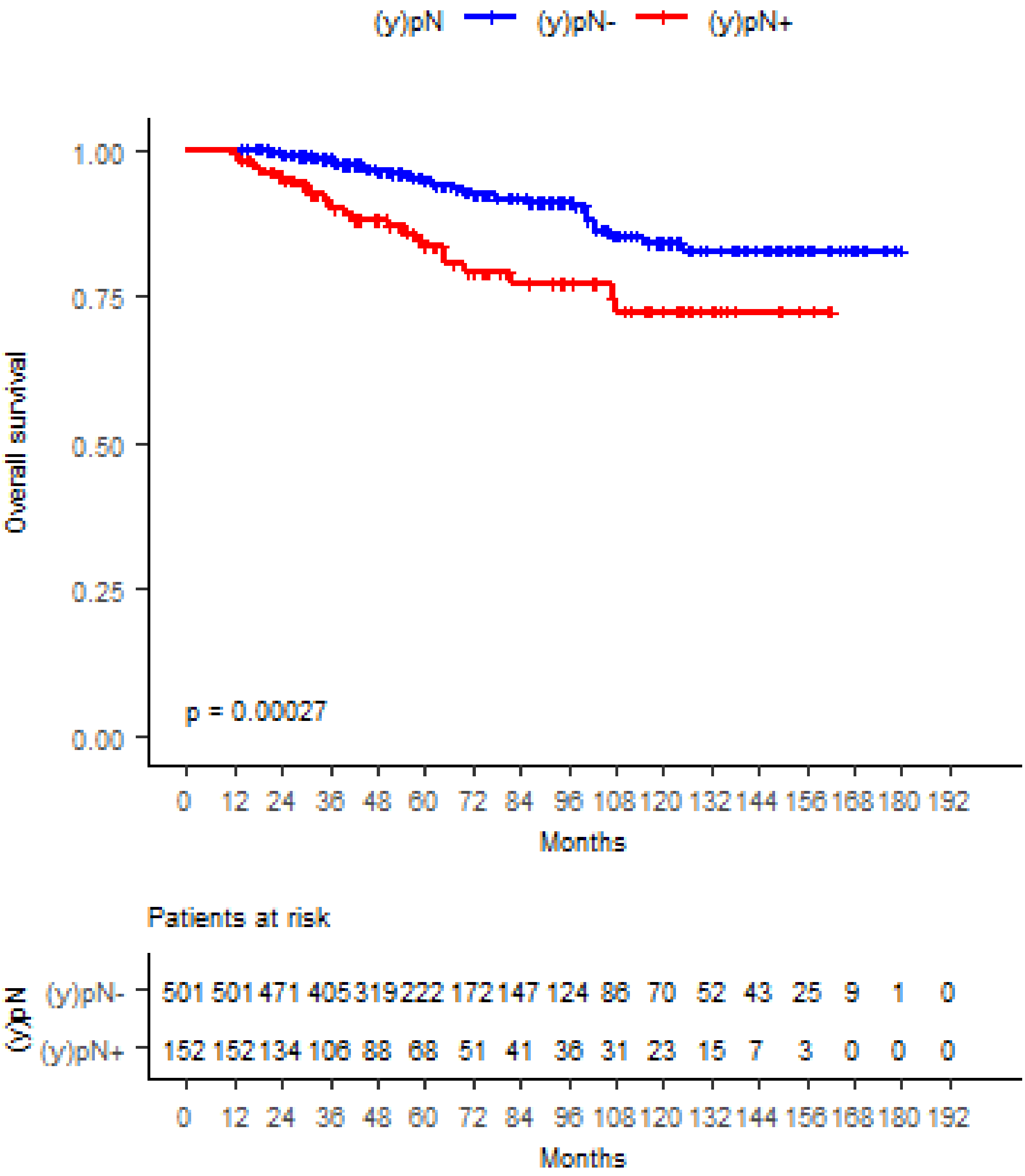

Figure 8. Overall survival according to grade.

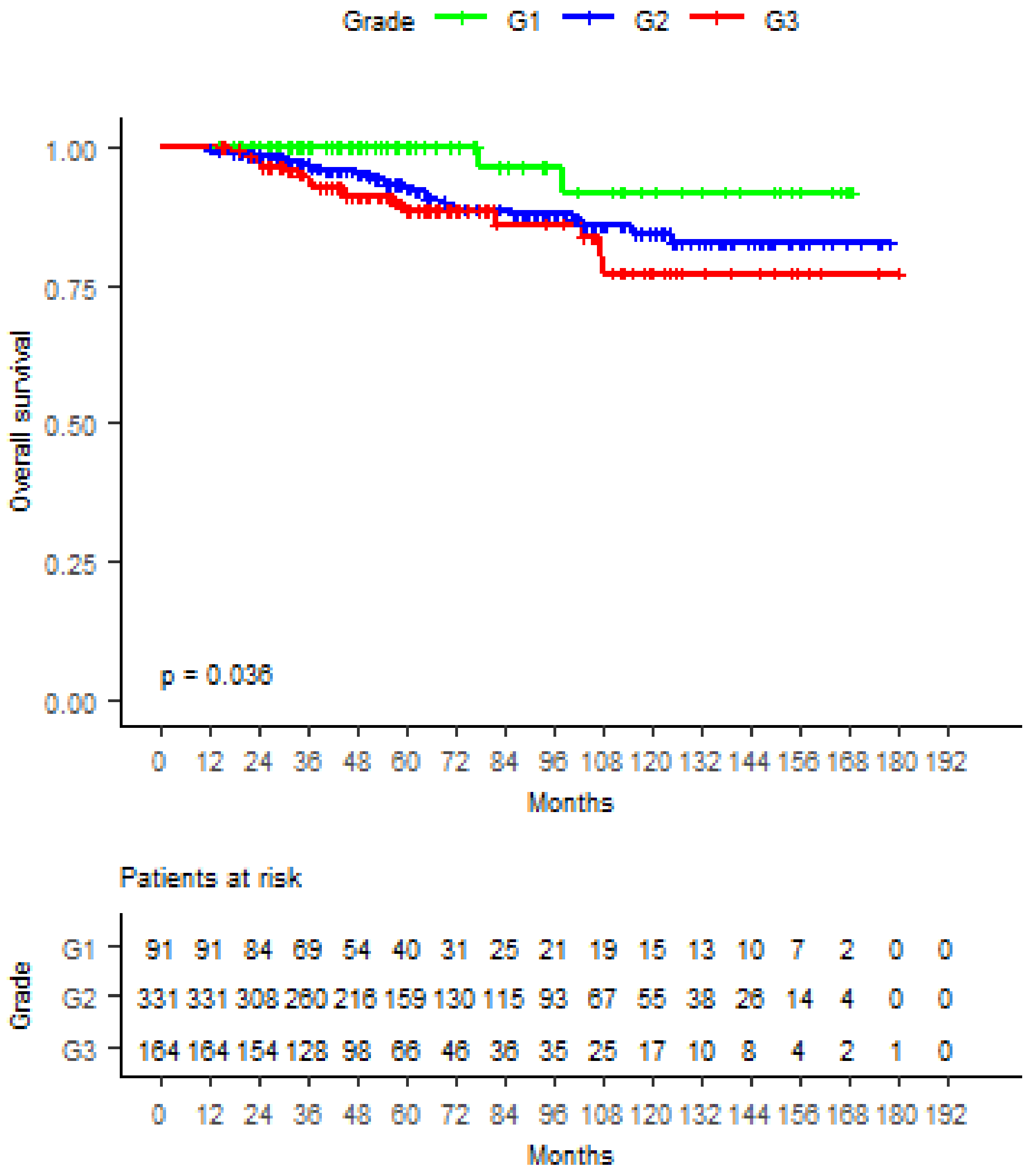

Figure 9. Overall survival according to estrogen receptors.

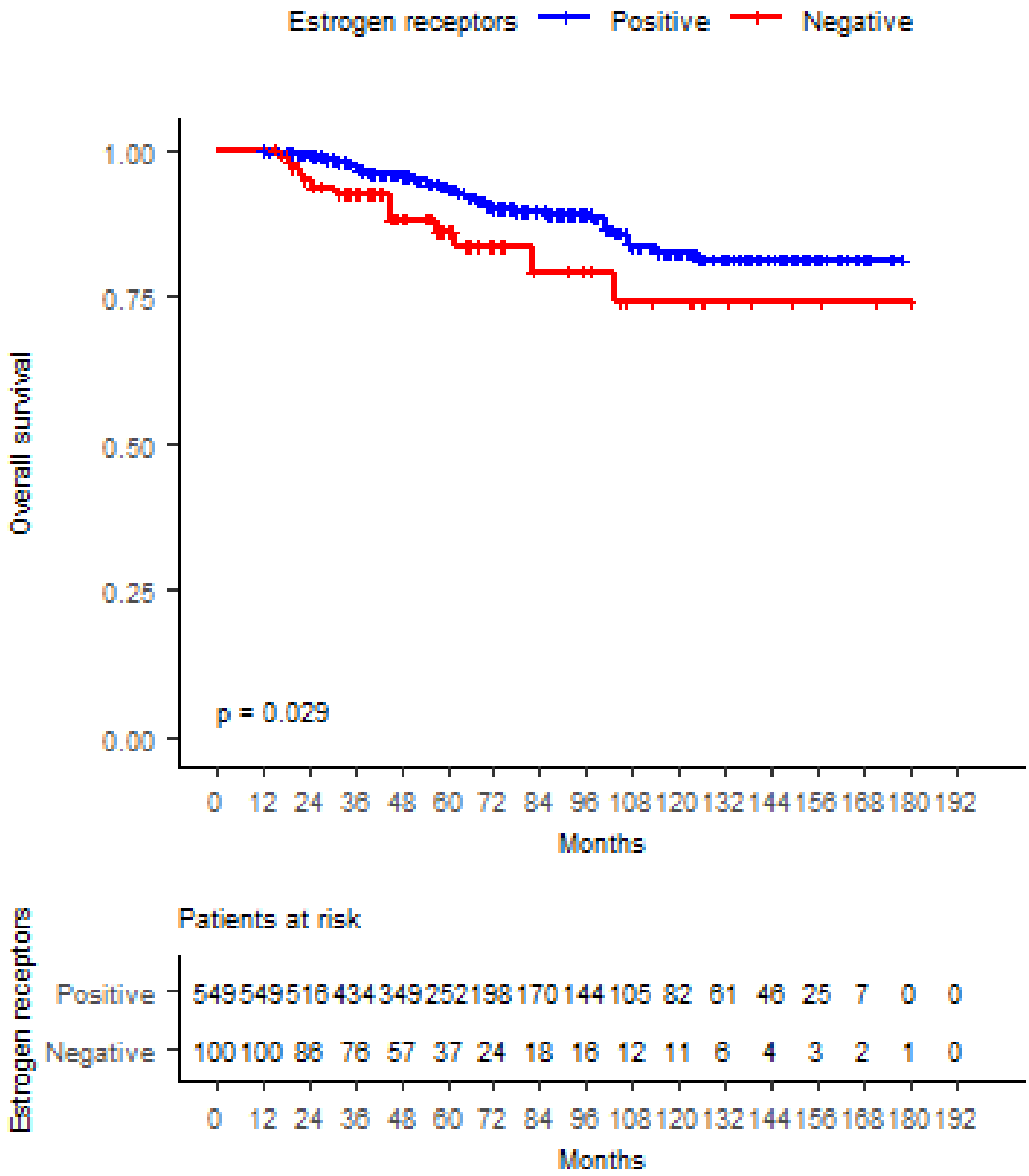

Figure 10. Overall survival according to progesterone receptors.

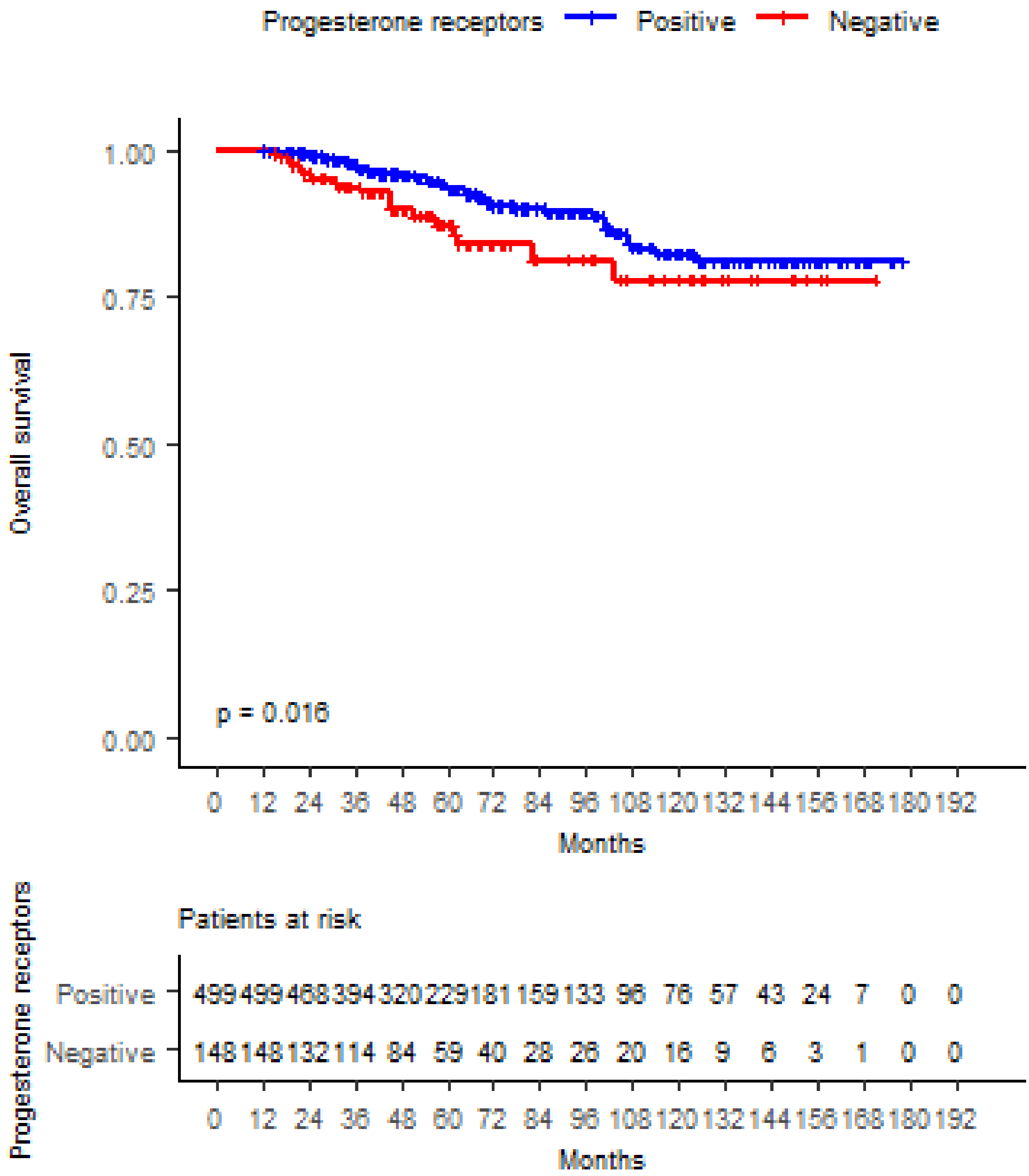

Table 2. Cox regression analysis of characteristics predicting time to death.

Characteristics	HR not adjusted	95% CI	p	HR adjusted*	95% CI	p
**cT**						
cT1	Ref.			Ref.		
cT2	1.25	0.73-2.15	0.413	1.03	0.53-2.01	0.923
cT3	3.57	1.38-9.26	**0.009**	3.55	1.16-10.88	**0.027**
**cN**						
cN0	Ref.					
cN1	2.13	1.27-3.56	**0.004**			
**Stage**						
I	Ref.			Ref.		
II	1.72	1.02-2.88	**0.041**	1.17	0.48-2.85	0.728
III	7.68	2.64-22.33	**<0.05**	2.92	0.61-13.94	0.179
**Hormone therapy**						
No	Ref.			Ref.		
Yes	0.52	0.29-0.94	**0.032**	0.66	0.27-1.60	0.359
**Adjuvant chemotherapy**						
No	Ref.			Ref.		
Yes	1.84	1.10-3.06	**0.019**	0.98	0.52-1.86	0.954
**(y)pN (evaluarlo)**						
(y)pN-	Ref.			Ref.		
(y)pN+	2.48	1.50-4.11	**<0.05**	3.22	1.04-9.94	**0.042**
**Grade**						
G1	Ref.					
G2	3.86	0.92-16.14	0.065			
G3	5.51	1.28-23.68	**0.022**			
**Estrogen receptors**						
Positive	Ref.			Ref.		
Negative	1.92	1.06-3.49	**0.032**	1.61	0.56-4.61	0.375
**Progesterone receptors**						
Positive	Ref.			Ref.		
Negative	1.74	1.01-2.99	**0.046**	1.06	0.481-2.342	0.883

* No included grade.

Table 3. Cumulative local recurrence rate by patient's characteristics.

	Cumulative local recurrence rate, %				
	N (events)	12m	60m	120m	p
**All patients**	653 (22)	0.3	2.3	7.9	
**Age, years**					
<50	159 (13)	0.6	7.7	12.9	
≥50	494 (9)	0.2	0.5	6.1	**<0.05**
**cT**					
cT1	430 (15)	0.5	1.9	7.6	
cT2	197 (5)	0.0	2.2	7.5	
cT3	26 (2)	0.0	8.5		**0.032**
**cN**					
cN0	519 (16)	0.2	1.5	8.0	
cN1	134 (6)	0.7	5.3	8.0	0.24
**Stage**					
I	372 (10)	0.0	0.9	6.5	
II	268 (11)	0.7	3.9	9.3	
III	13 (1)	0.0	8.3		**0.031**
**Frozen section before IORT?**					
Yes	596 (21)	0.3	2.2	7.8	
No	53 (1)	0.0	2.2		0.39
**Was margin extension performed after IORT?**					
Yes	151 (6)	0.0	1.4	13.2	
No	495 (16)	0.4	2.6	6.6	0.61
**Final paraffin situation**					
R0	600 (20)	0.3	2.2	7.2	
R1	52 (2)	0.0	3.2	27.4	0.54
**Applicator diameter, cm**					
≤3	100 (4)	0.0	4.2	7.1	

≥3.5	509 (13)	0.4	1.9	4.7	0.2
**Hormone therapy**					
No	105 (6)	0.0	5.0	13.9	
Yes	548 (16)	0.4	1.9	7.1	**0.034**
**Adjuvant chemotherapy**					
No	348 (10)	0.0	0.4	7.4	
Yes	305 (12)	0.7	4.5	6.6	0.071
**Fractionation**					
Hypofractionation (>2Gy/Fx)	159 (1)	0.0	1.8		
Normofractionation (1.8-2Gy/Fx)	492 (21)	0.4	2.5	8.1	0.34
**(y)pT**					
(y)pT0	28 (1)	0.0	9.1		
(y)pT1	435 (15)	0.5	1.7	7.4	
(y)pT2	190 (6)	0.0	2.9	8.3	0.89
**(y)pN**					
(y)pN(-)	501 (14)	0.0	1.1	7.8	
(y)pN(+)	152 (8)	1.3	6.2	8.8	**0.003**
**Grade**					
G1	91 (1)	0.0	0.0	4.2	
G2	331 (13)	0.6	2.2	8.5	
G3	164 (7)	0.0	4.8	10.1	0.33
**In situ component**					
Yes	131 (4)	0.0	3.9	17.6	
No	468 (16)	0.4	2.3	6.9	0.23
**Margins from in situ component, mm**					
≥2	239 (8)	0.4	1.7	4.5	
<2	162 (5)	0.0	3.8	8.6	0.4
**Estrogen receptors**					
Positive	549 (19)	0.4	2.1	8.4	
Negative	100 (3)	0.0	3.1	3.1	0.88

**Progesterone receptors**					
Positive	499 (17)	0.2	2.2	8.5	
Negative	148 (5)	0.7	2.8	4.7	0.69
**HER2**					
Positive	105 (3)	0.0	1.3	8.8	
Negative	540 (19)	0.4	2.5	7.7	0.75
**Subtype**					
Triple negative	69 (3)	0.0	4.7	4.7	
Luminal A	395 (13)	0.3	2.0	7.1	
Luminal B	81 (3)	1.2	2.9		
HER2,Luminal/HER2	105 (3)	0.0	1.3	8.8	0.3

Figure 11. Cumulative local recurrence rate.

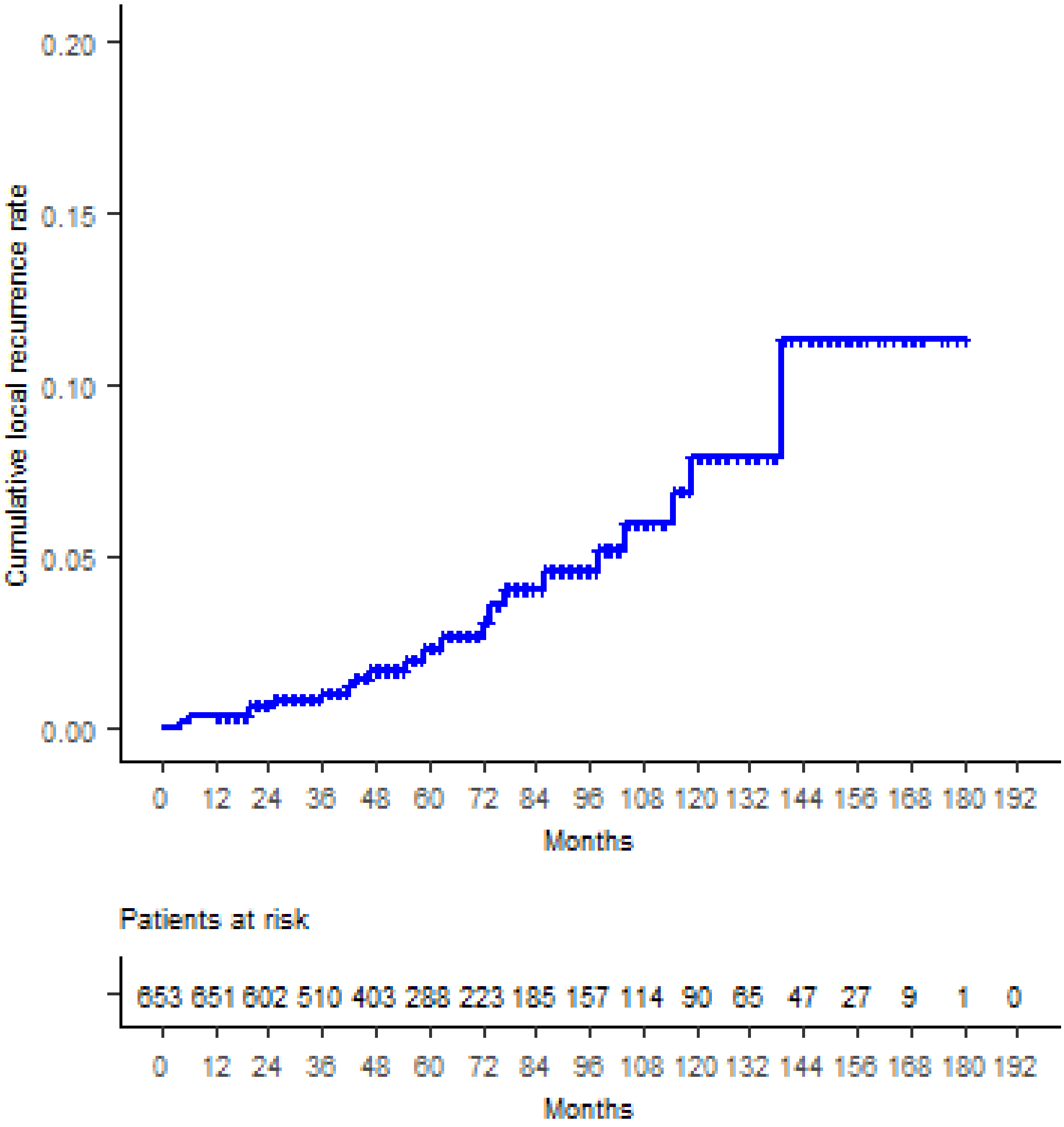

Figure 12. Cumulative local recurrence rate according age.

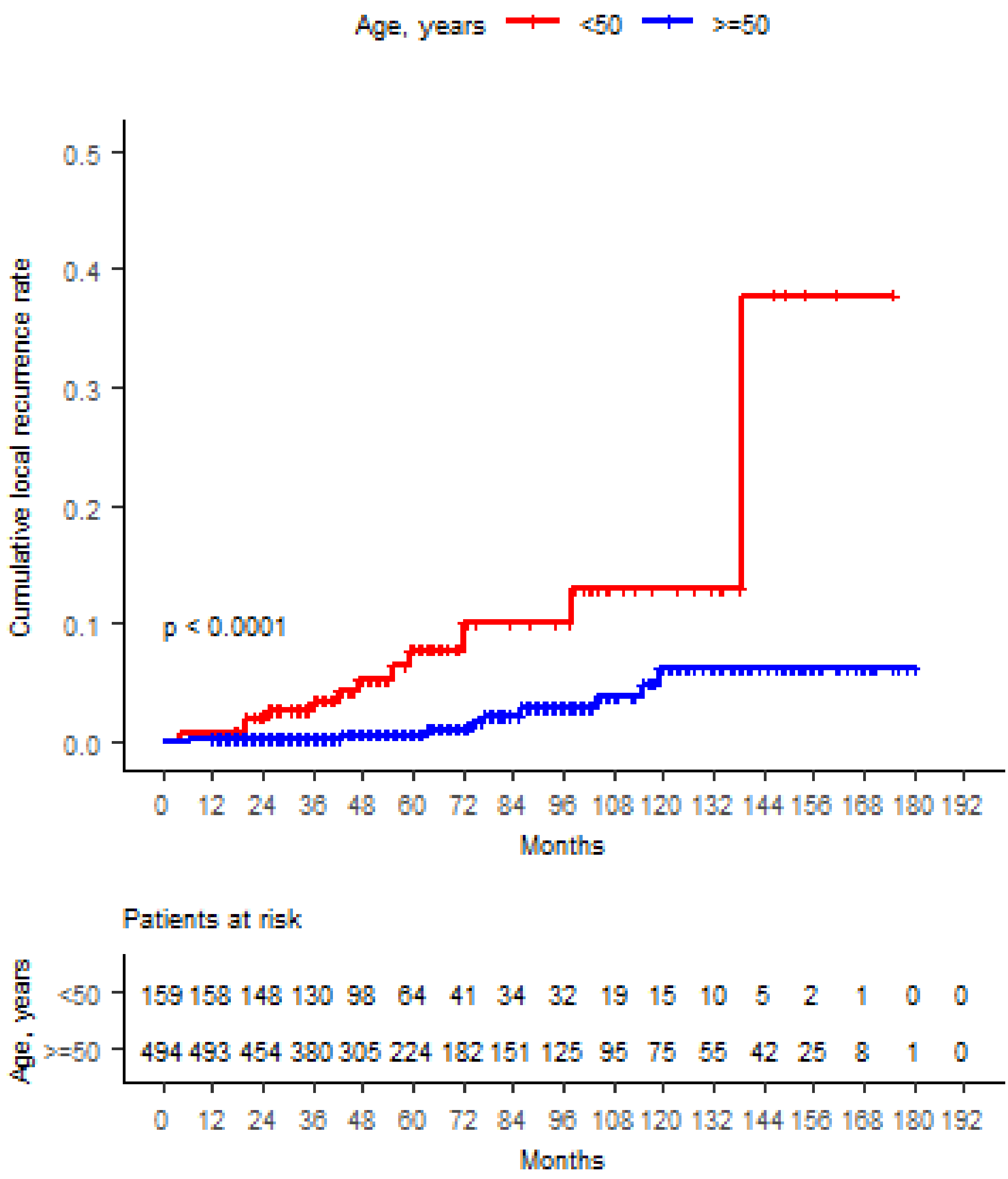

Figure 13. Cumulative local recurrence rate according cT.

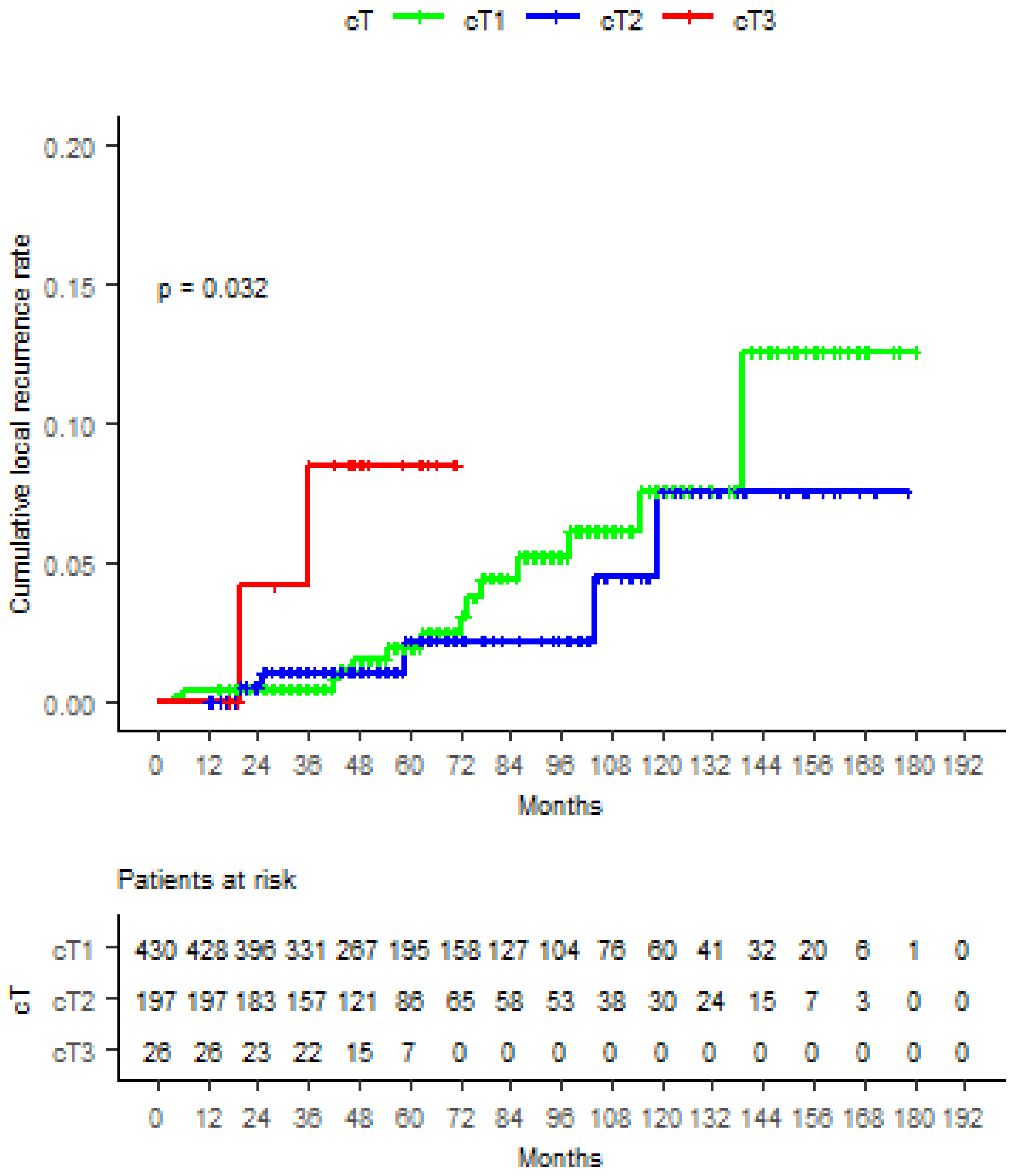

Figure 14. Cumulative local recurrence rate according stage.

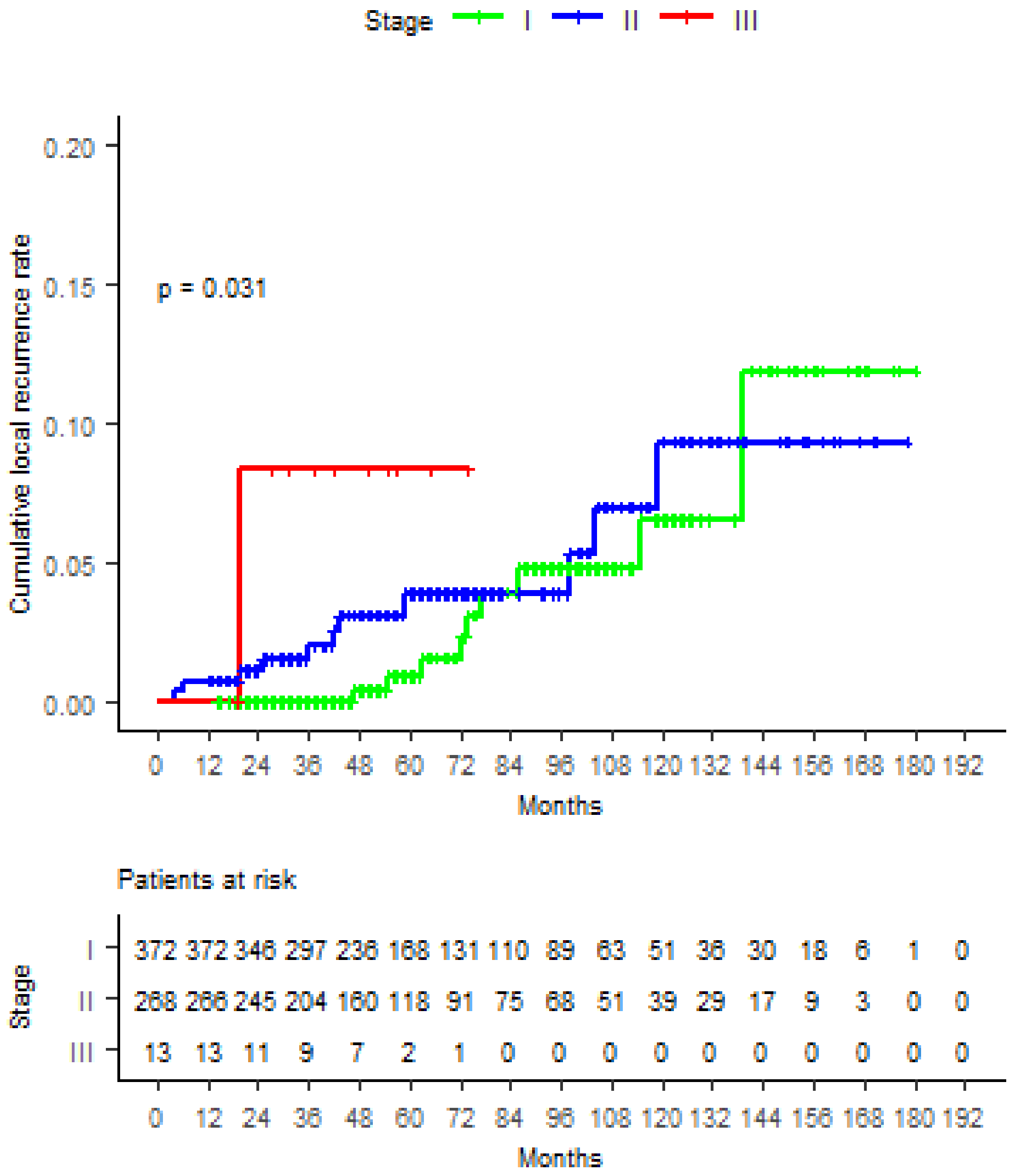

Figure 15. Cumulative local recurrence rate with and without hormone therapy.

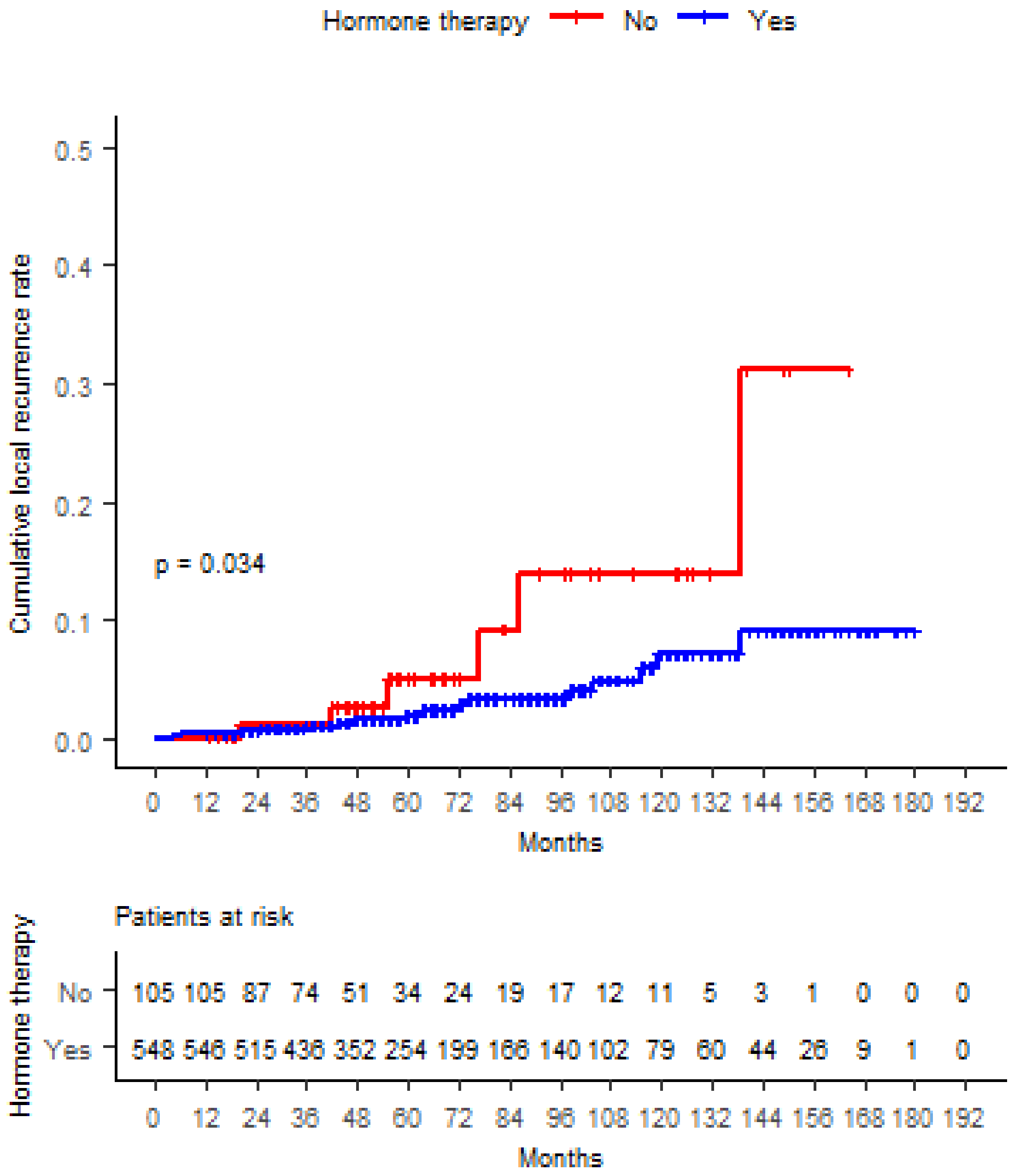

Figure 16. Cumulative local recurrence rate according to pN.

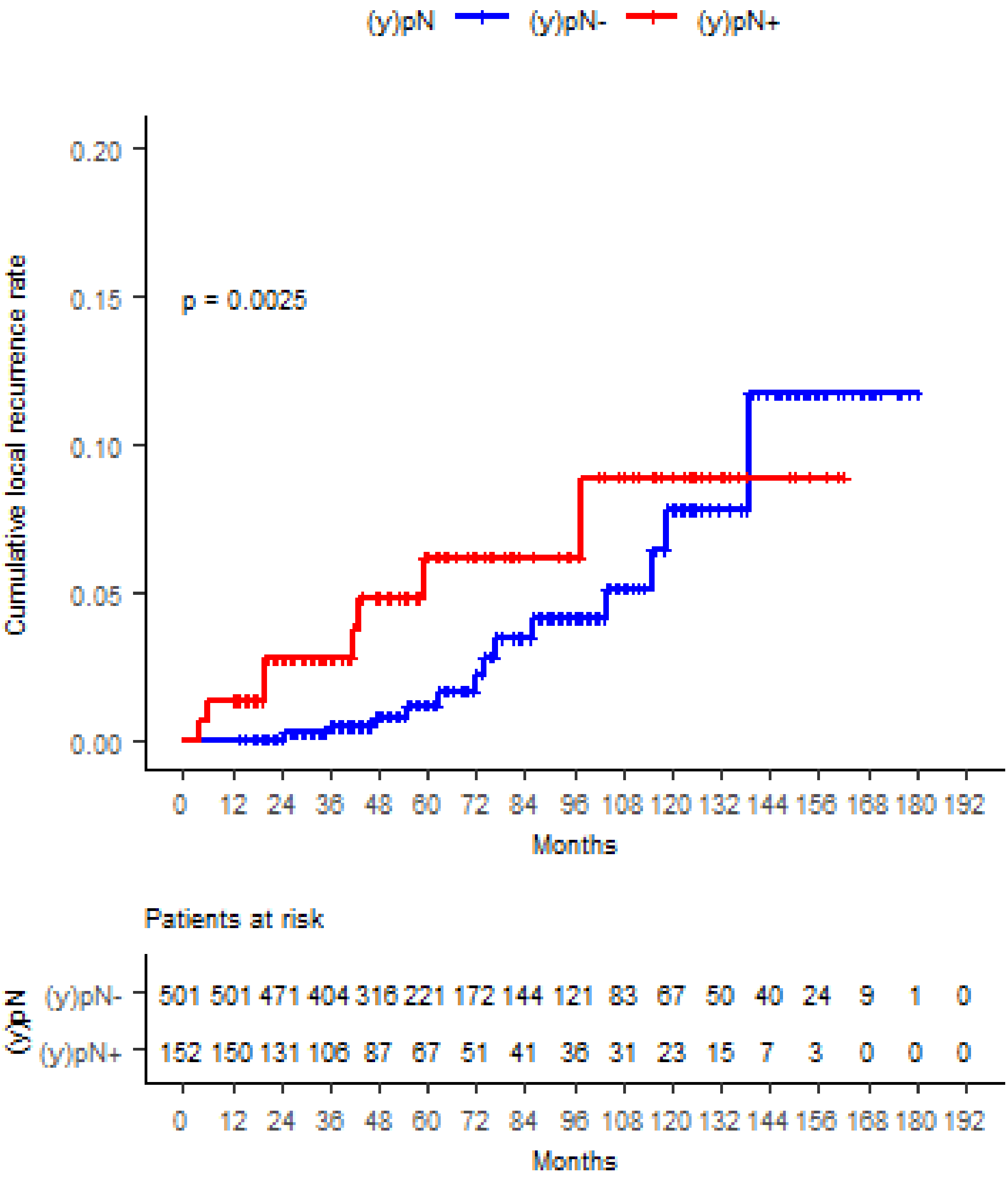

Table 4. Cox regression analysis of characteristics predicting time to local recurrence.

Characteristics	HR not adjusted	95% CI	p	HR adjusted	95% CI	p
**Age, years**						
<50	Ref.			Ref.		
≥50	0.18	0.07-0.41	**<0.05**	0.19	0.08-0.47	**<0.05**
**cT**						
cT1	Ref.			Ref.		
cT2	0.72	0.26-1.97	0.518	0.63	0.23-1.75	0.379
cT3	4.84	1.03-22.66	**0.045**	2.29	0.48-10.99	0.300
**Stage**						
I	Ref.					
II	1.57	0.67-3.71	0.300			
III	6.46	0.80-52.47	0.081			
**Hormone therapy**						
No	Ref.			Ref.		
Yes	0.37	0.15-0.96	**0.041**	0.47	0.18-1.21	0.117
**(y)pN**						
(y)pN-	Ref.					
(y)pN+	2.05	0.85-4.89	0.108			

Table 5. Cumulative regional recurrence rate by patient's characteristics.

		Cumulative regional recurrence rate, %			
	N (events)	12m	60m	120m	p
**All patients**	653 (9)	0.5	1.2	2.1	
**Age, years**					
<50	159 (4)	0.6	2.9	2.9	
≥50	494 (5)	0.4	0.7	1.7	0.1
**cT**					
cT1	430 (4)	0.5	0.8	1.4	
cT2	197 (4)	0.5	1.7	3.2	
cT3	26 (1)	0.0	4.2		0.24
**cN**					
cN0	519 (6)	0.4	0.8	1.9	
cN1	134 (3)	0.7	2.7	2.7	0.28
**Stage**					
I	372 (3)	0.0	0.9	6.5	
II	268 (6)	0.7	3.9	9.3	
III	13 (0)	0.0	8.3		0.28
**Frozen section before IORT?**					
Yes	596 (9)	0.5	1.3	2.2	
No	53 (0)	0.0	0.0		0.5
**Was margin extension performed after IORT?**					
Yes	151 (3)	0.7	1.4	3.3	
No	495 (6)	0.4	1.2	1.7	0.5
**Final paraffin situation**					
R0	600 (8)	0.5	1.1	1.9	
R1	52 (1)	0.0	3.6	3.6	0.58
**Applicator diameter, cm**					
≤3	100 (1)	0.0	1.0	1.0	

≥3.5	509 (7)	0.6	1.0	2.2	0.83
**Hormone therapy**					
No	105 (2)	0.0	1.8	6.1	
Yes	548 (7)	0.5	1.1	1.6	0.5
**Adjuvant chemotherapy**					
No	348 (2)	0.0	0.0	1.3	
Yes	305 (7)	1.0	2.6	2.6	**0.038**
**Fractionation**					
Hypofractionation (>2Gy/Fx)	159 (0)	0.0	0.0		
Normofractionation (1.8-2Gy/Fx)	492 (9)	0.6	1.5	2.4	0.16
**(y)pT**					
(y)pT0	28 (0)	0.0	0.0		
(y)pT1	435 (5)	0.5	1.1	1.6	
(y)pT2	190 (4)	0.5	1.7	3.4	0.54
**(y)pN**					
(y)pN(-)	501 (6)	0.2	0.9	2.0	
(y)pN(+)	152 (3)	1.3	2.4	2.4	0.35
**Grade**					
G1	91 (1)	1.1	1.1	1.1	
G2	331 (5)	0.6	1.4	2.1	
G3	164 (2)	0.0	0.7	3.0	0.96
**In situ component**					
Yes	131 (1)	0.0	0.9	0.9	
No	468 (8)	0.6	1.4	2.5	0.61
**Margins from in situ component, mm**					
≥2	239 (3)	0.0	0.5	2.3	
<2	162 (4)	1.2	3.0	3.0	0.14
**Estrogen receptors**					
Positive	549 (7)	0.5	1.2	1.6	
Negative	100 (2)	0.0	1.2	6.2	0.47

**Progesterone receptors**					
Positive	499 (7)	0.6	1.3	1.8	
Negative	148 (2)	0.0	0.8	4.0	0.94
**HER2**					
Positive	105 (2)	0.0	2.1	2.1	
Negative	540 (7)	0.6	1.0	2.1	0.54
**Subtype**					
Triple negative	69 (1)	100.0	100.0	92.3	
Luminal A	395 (5)	99.5	98.8	98.3	
Luminal B	81 (1)	98.8	98.8		
HER2,Luminal/HER2	105 (2)	100.0	97.9	97.9	0.92

Figure 17. Cumulative regional recurrence rate.

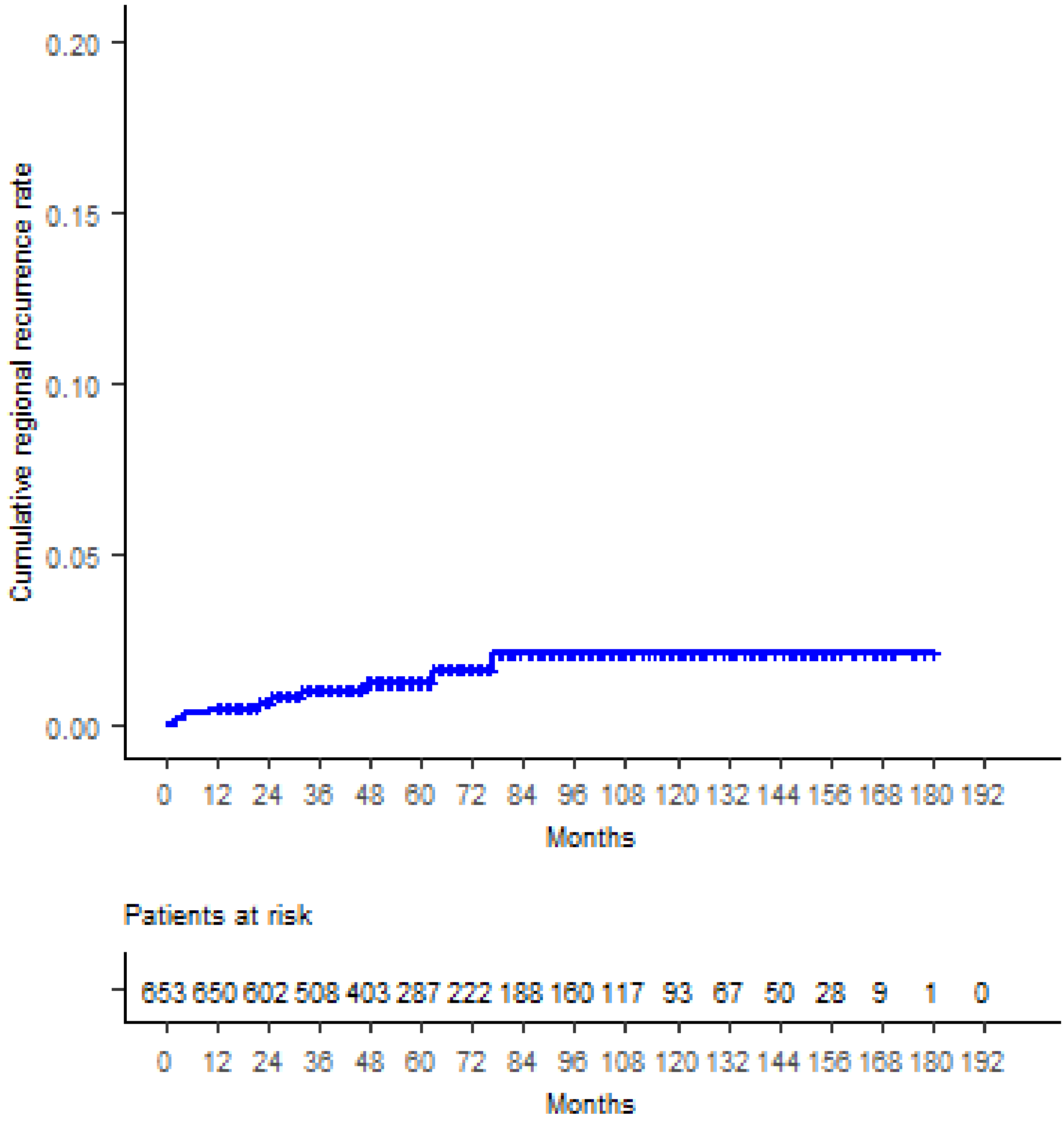

Figure 18. Cumulative regional recurrence rate with and without adjuvant chemotherapy.

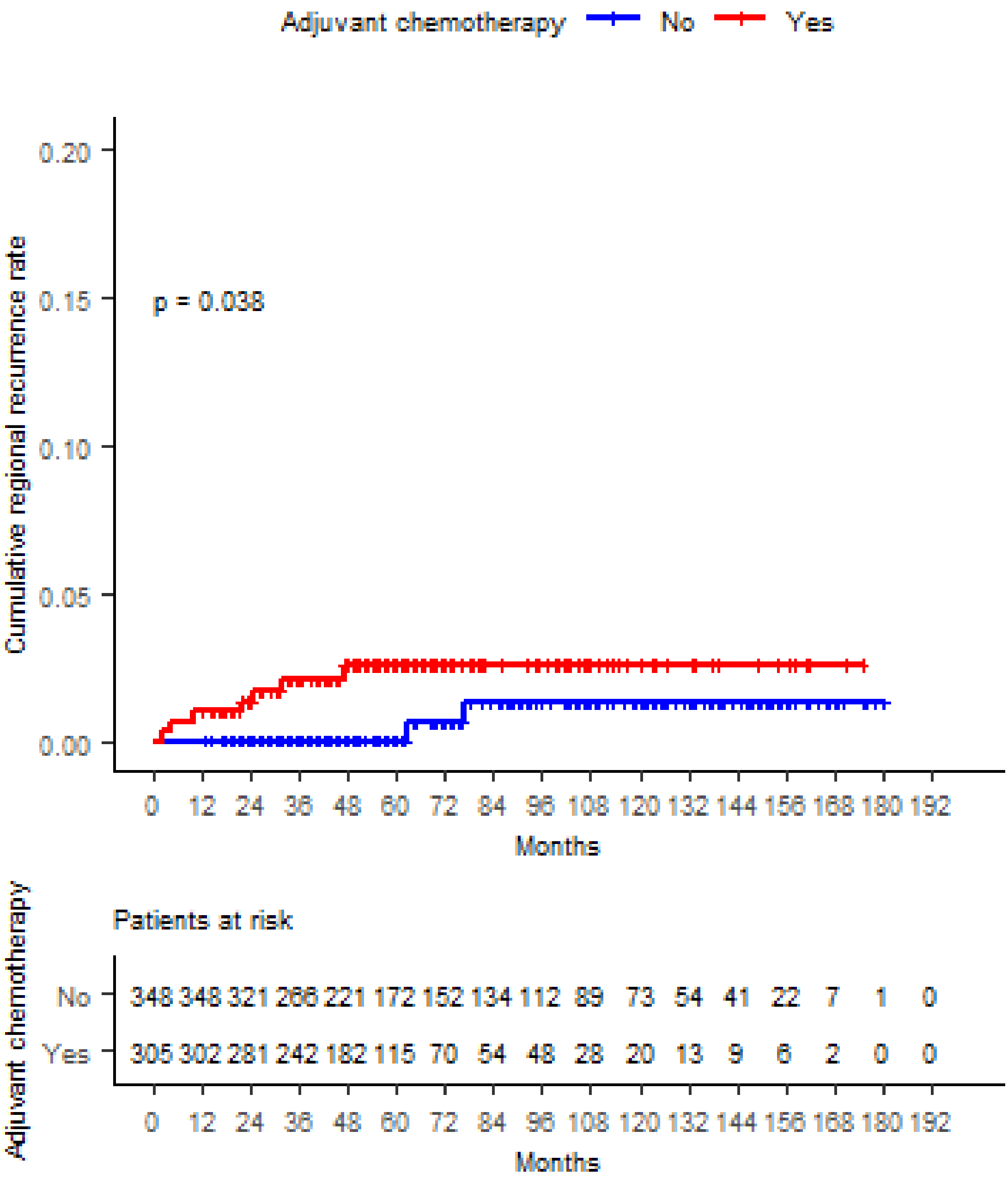

Table 6. Cumulative distant recurrence rate by patient's characteristics.

	Cumulative distant recurrence rate, %				
	N (events)	12m	60m	120m	p
**All patients**	653 (45)	0.6	6.9	13.2	
**Age, years**					
<50	159 (13)	0.0	7.8	18.1	
≥50	494 (32)	0.8	6.4	12.0	0.36
**cT**					
cT1	430 (25)	0.2	5.5	11.5	
cT2	197 (16)	1.5	8.1	15.3	
cT3	26 (4)	0.0	16.4		**0.032**
**cN**					
cN0	519 (24)	0.0	4.8	10.3	
cN1	134 (21)	3.0	14.7	23.1	**<0.05**
**Stage**					
I	372 (15)	0.0	4.1	8.8	
II	268 (28)	1.5	10.1	18.7	
III	13 (2)	0.0	17.9		**0.001**
**Frozen section before IORT?**					
Yes	596 (43)	0.7	6.8	13.1	
No	53 (1)	0.0	2.0		0.79
**Was margin extension performed after IORT?**					
Yes	151 (8)	0.0	7.7	7.7	
No	495 (37)	0.8	6.6	14.6	0.37
**Final paraffin situation**					
R0	600 (40)	0.7	6.3	12.8	
R1	52 (5)	0.0	15.2	15.2	0.11
**Applicator diameter, cm**					
≤3	100 (7)	0.0	8.8	8.8	

≥3.5	509 (33)	0.8	6.4	13.4	0.58
**Hormone therapy**					
No	105 (7)	1.0	6.1	14.4	
Yes	548 (38)	0.5	6.9	13.0	0.69
**Adjuvant chemotherapy**					
No	348 (14)	0.0	4.1	7.5	
Yes	305 (31)	1.3	10.0	23.7	**<0.05**
**Fractionation**					
Hypofractionation (>2Gy/Fx)	159 (1)	0.0	2.6		
Normofractionation (1.8-2Gy/Fx)	492 (44)	0.8	7.9	14.2	**0.021**
**(y)pT**					
(y)pT0	28 (1)	0.0	3.6		
(y)pT1	435 (28)	0.2	6.3	12.1	
(y)pT2	190 (16)	1.6	8.5	15.9	0.65
**(y)pN**					
(y)pN(-)	501 (23)	0.0	4.6	10.1	
(y)pN(+)	152 (22)	2.6	14.8	23.2	**<0.05**
**Grade**					
G1	91 (1)	0.0	2.5	2.5	
G2	331 (23)	0.9	6.9	12.3	
G3	164 (12)	0.6	7.0	15.6	0.1
**In situ component**					
Yes	131 (5)	0.0	6.0	10.5	
No	468 (37)	0.9	7.8	13.9	0.55
**Margins from in situ component, mm**					
≥2	239 (22)	1.3	9.1	15.9	
<2	162 (10)	0.6	8.1	8.1	0.64
**Estrogen receptors**					
Positive	549 (38)	0.5	6.6	13.2	
Negative	100 (7)	1.0	8.8	11.9	0.68

**Progesterone receptors**					
Positive	499 (32)	0.4	6.3	11.8	
Negative	148 (13)	1.4	9.2	19.1	0.18
**HER2**					
Positive	105 (15)	1.9	14.4	26.3	
Negative	540 (30)	0.4	5.3	10.6	**0.002**
**Subtype**					
Triple negative	69 (3)	0.0	4.0	9.1	
Luminal A	395 (22)	0.5	4.8	10.0	
Luminal B	81 (5)	0.0	9.4		
HER2,Luminal/HER2	105 (15)	1.9	14.3	26.2	**0.011**

Figure 19. Cumulative distant recurrence rate.

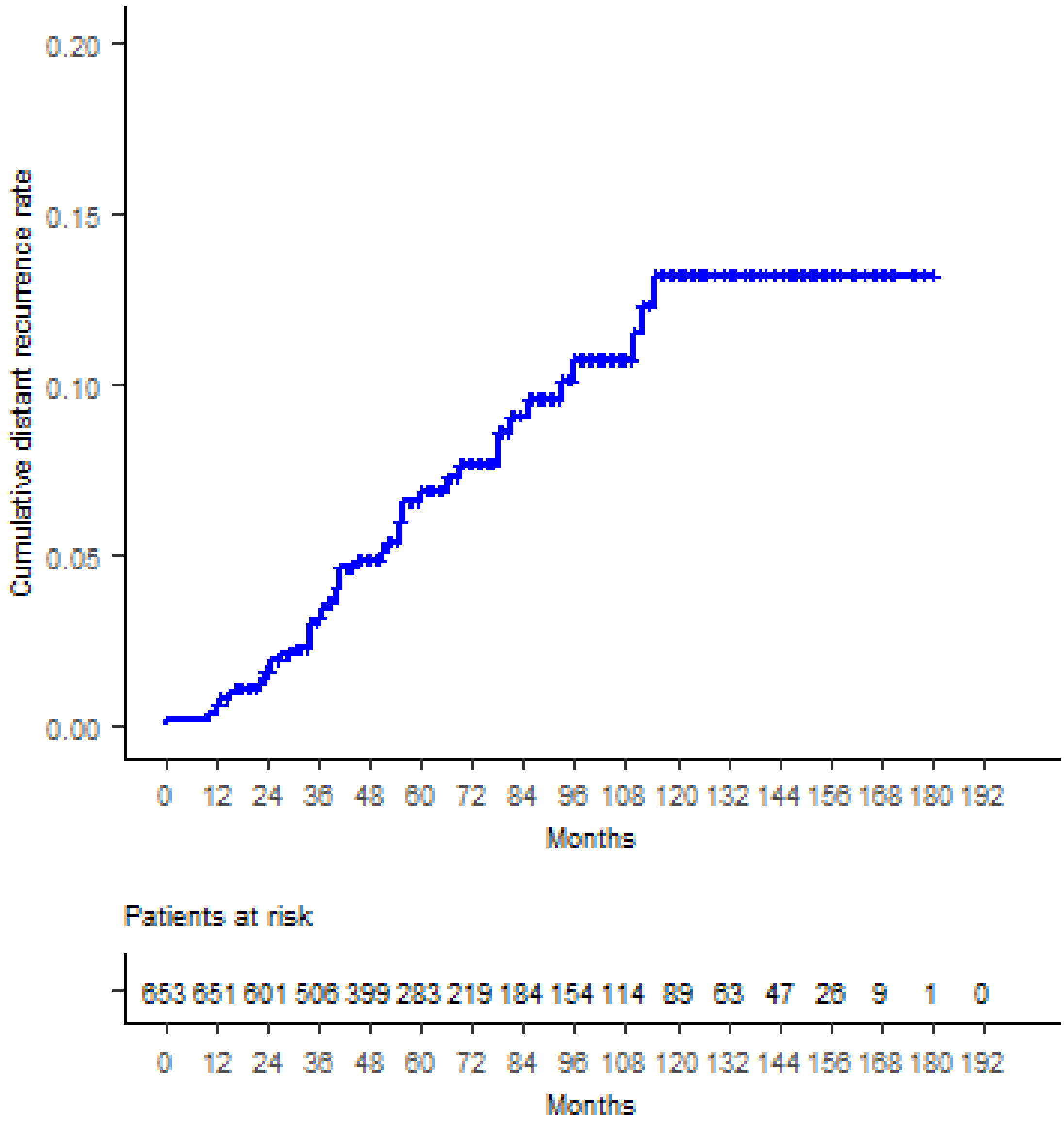

Figure 20. Cumulative distant recurrence rate according to cT.

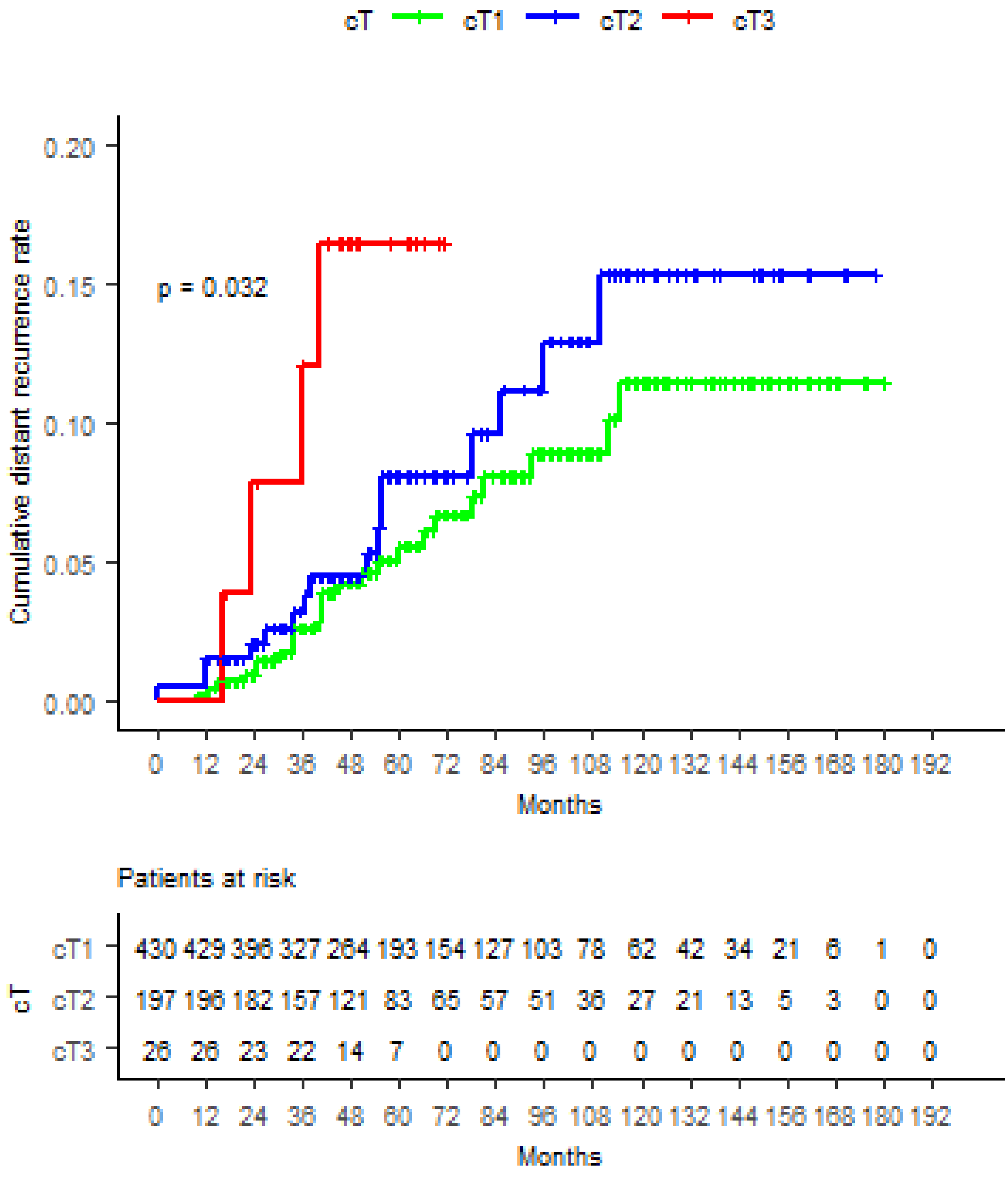

Figure 21. Cumulative distant recurrence rate according to cN.

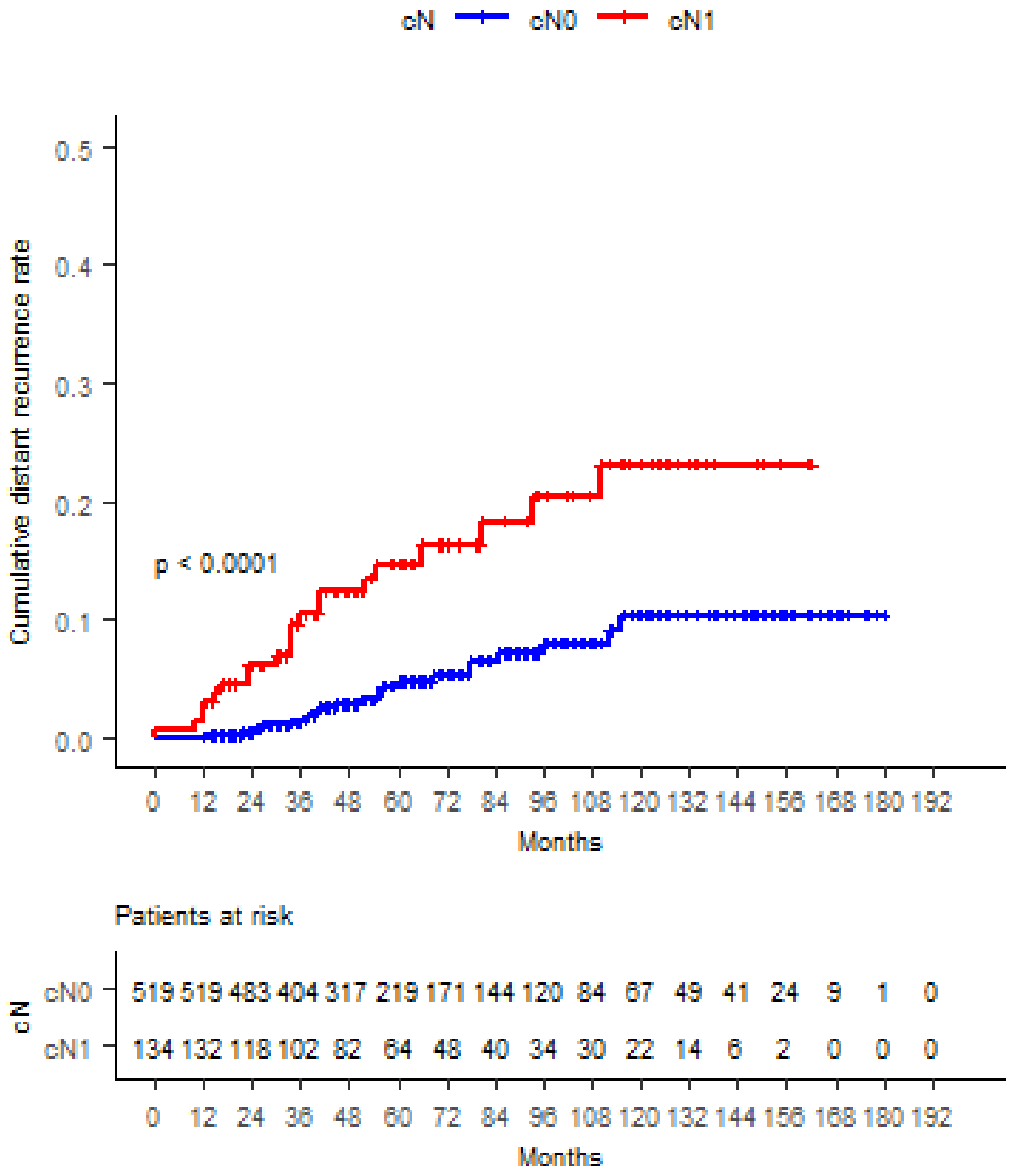

Figure 22. Cumulative distant recurrence rate according to stage.

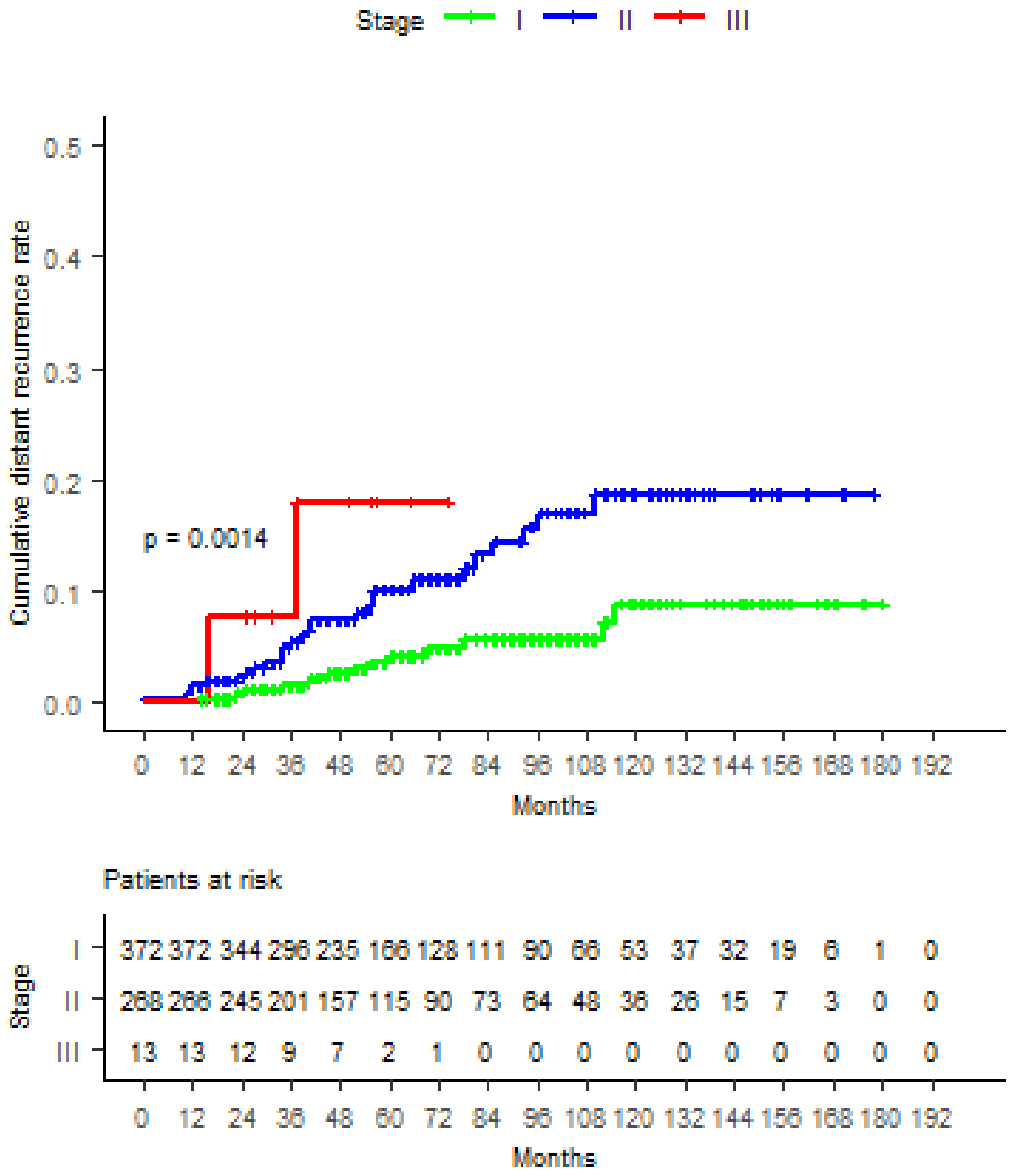

Figure 23. Cumulative distant recurrence rate with and without adjuvant chemotherapy.

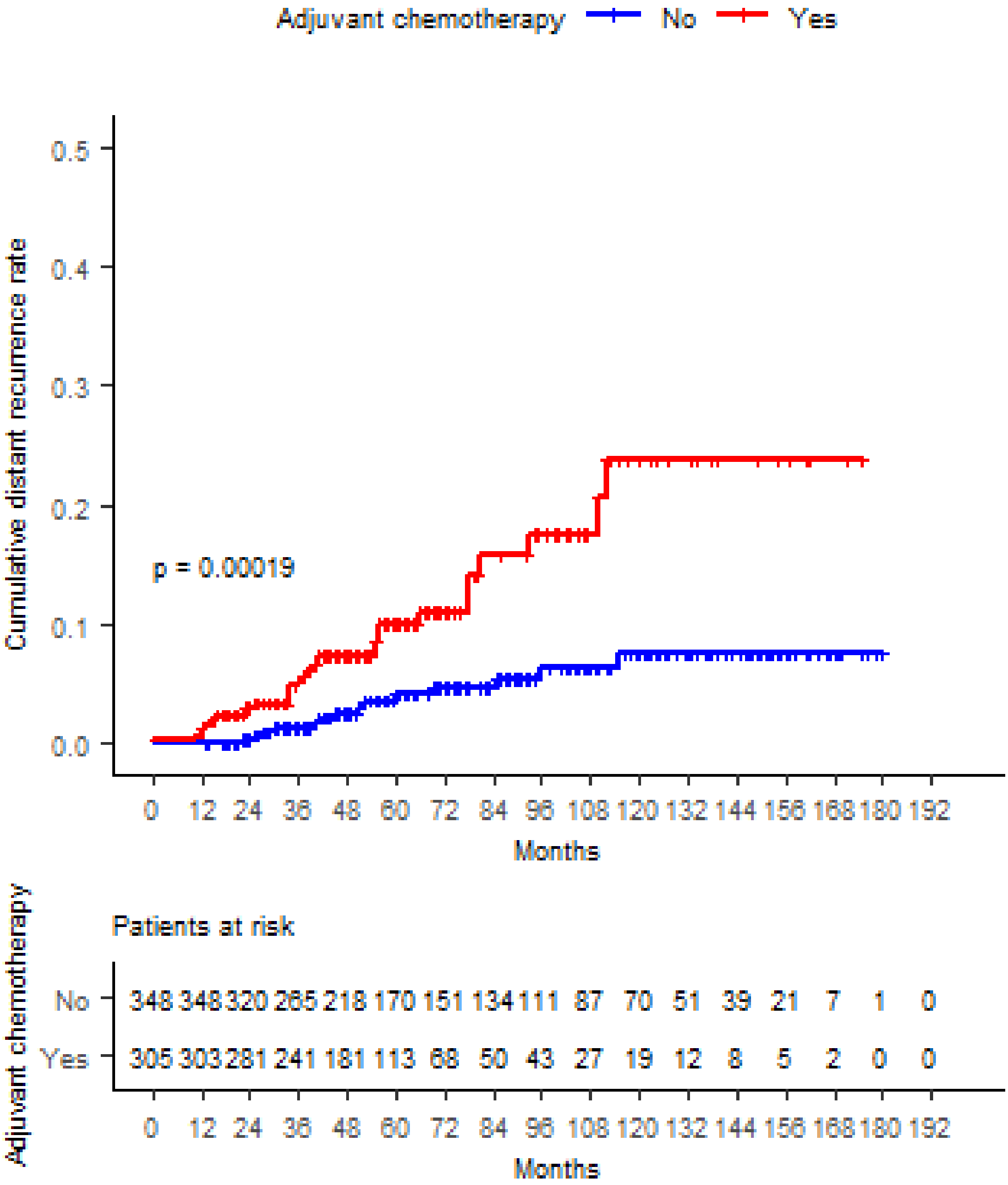

Figure 24. Cumulative distant recurrence rate according to fractionation.

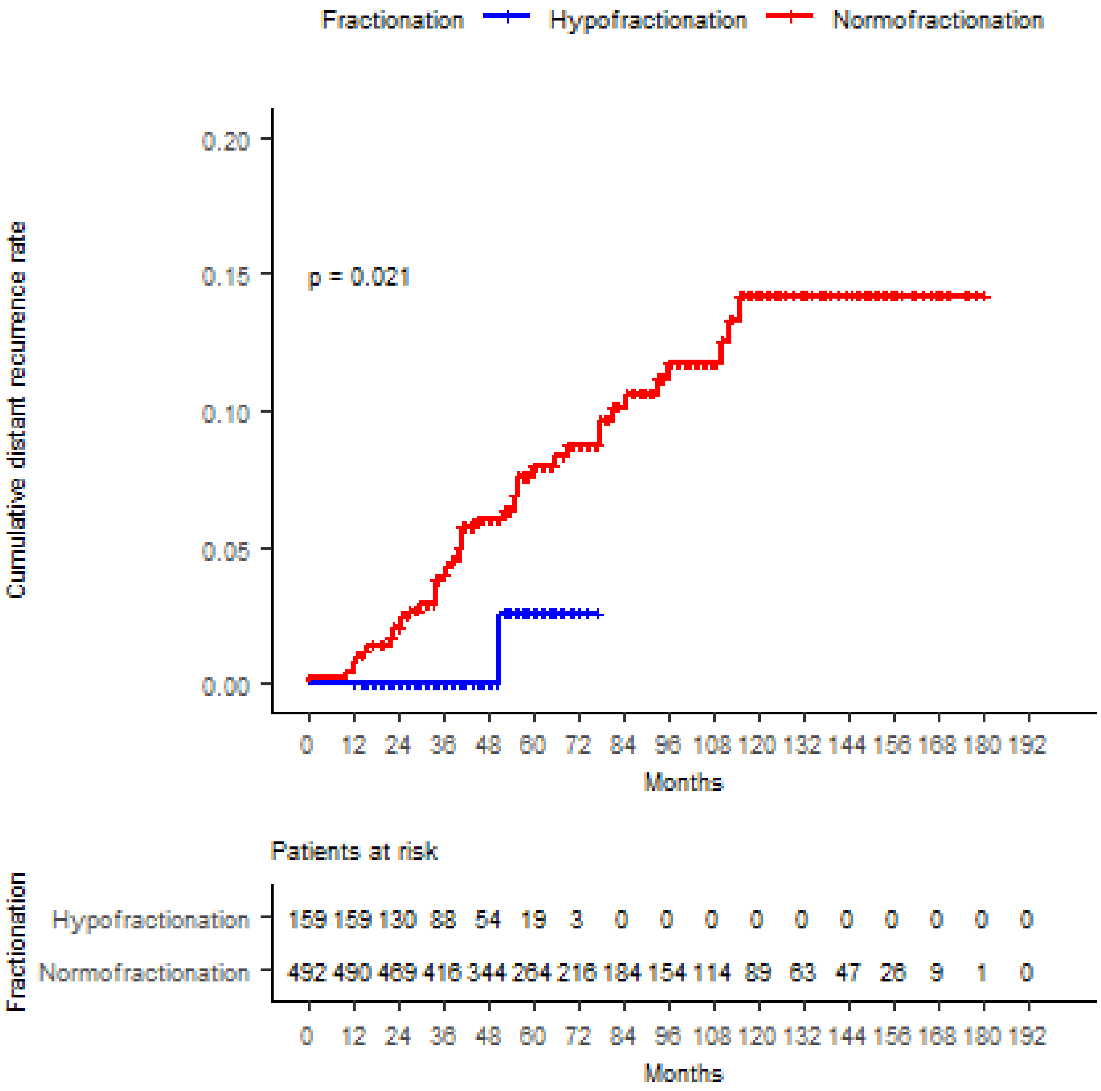

Figure 25. Cumulative distant recurrence rate according to pN.

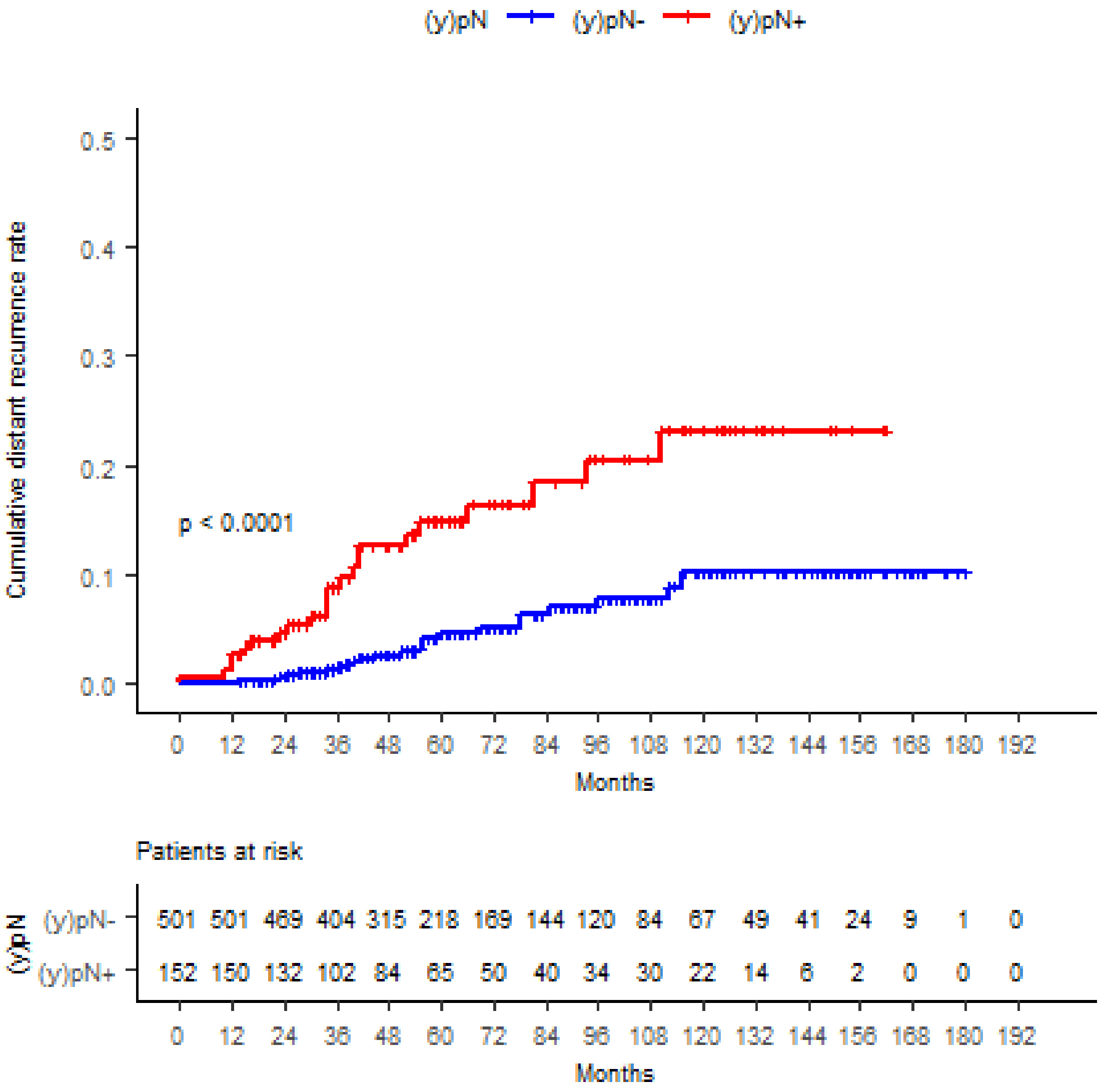

Figure 26. Cumulative distant recurrence rate according to HER2.

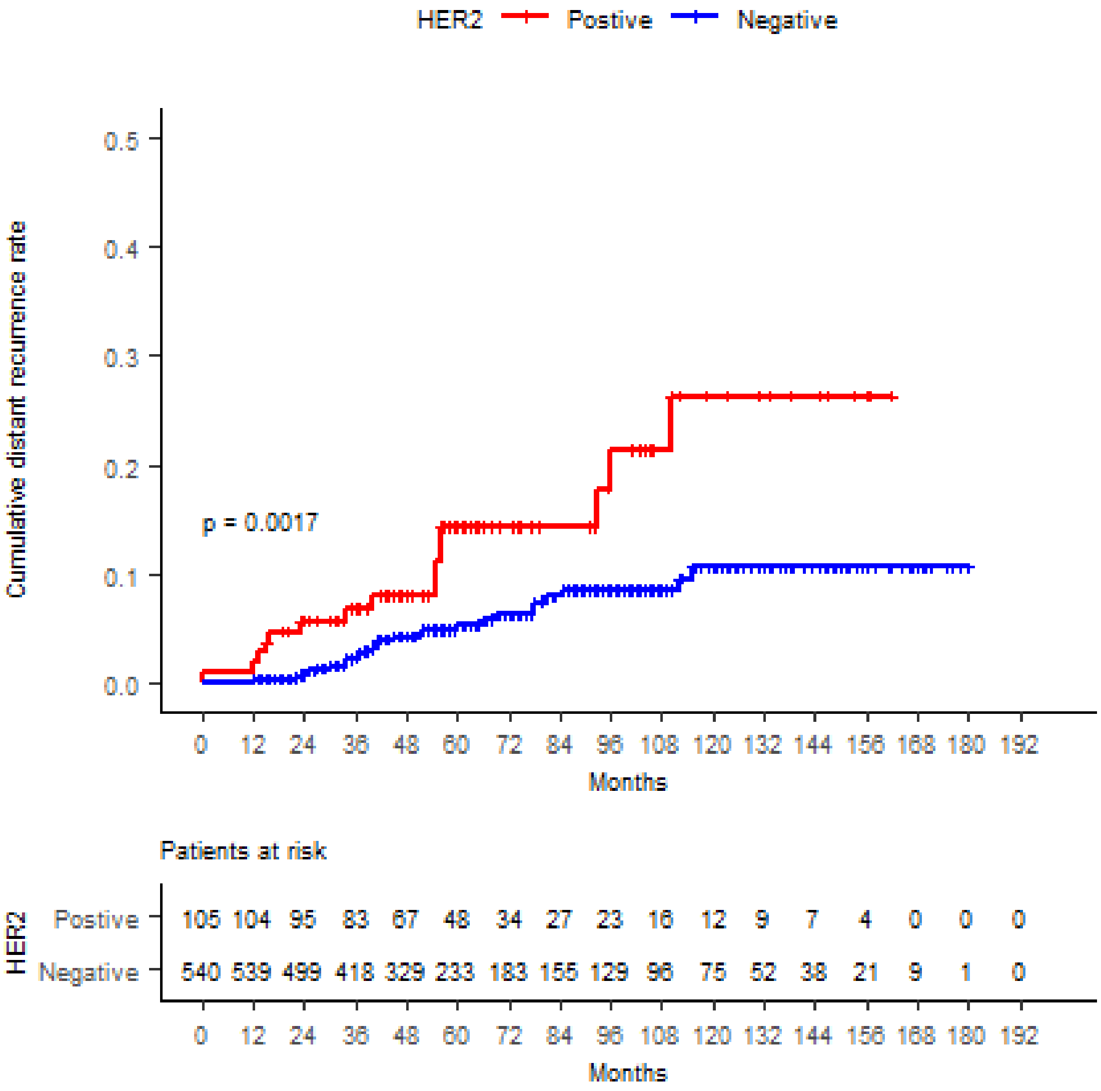

Figure 27. Cumulative distant recurrence rate according to subtype.

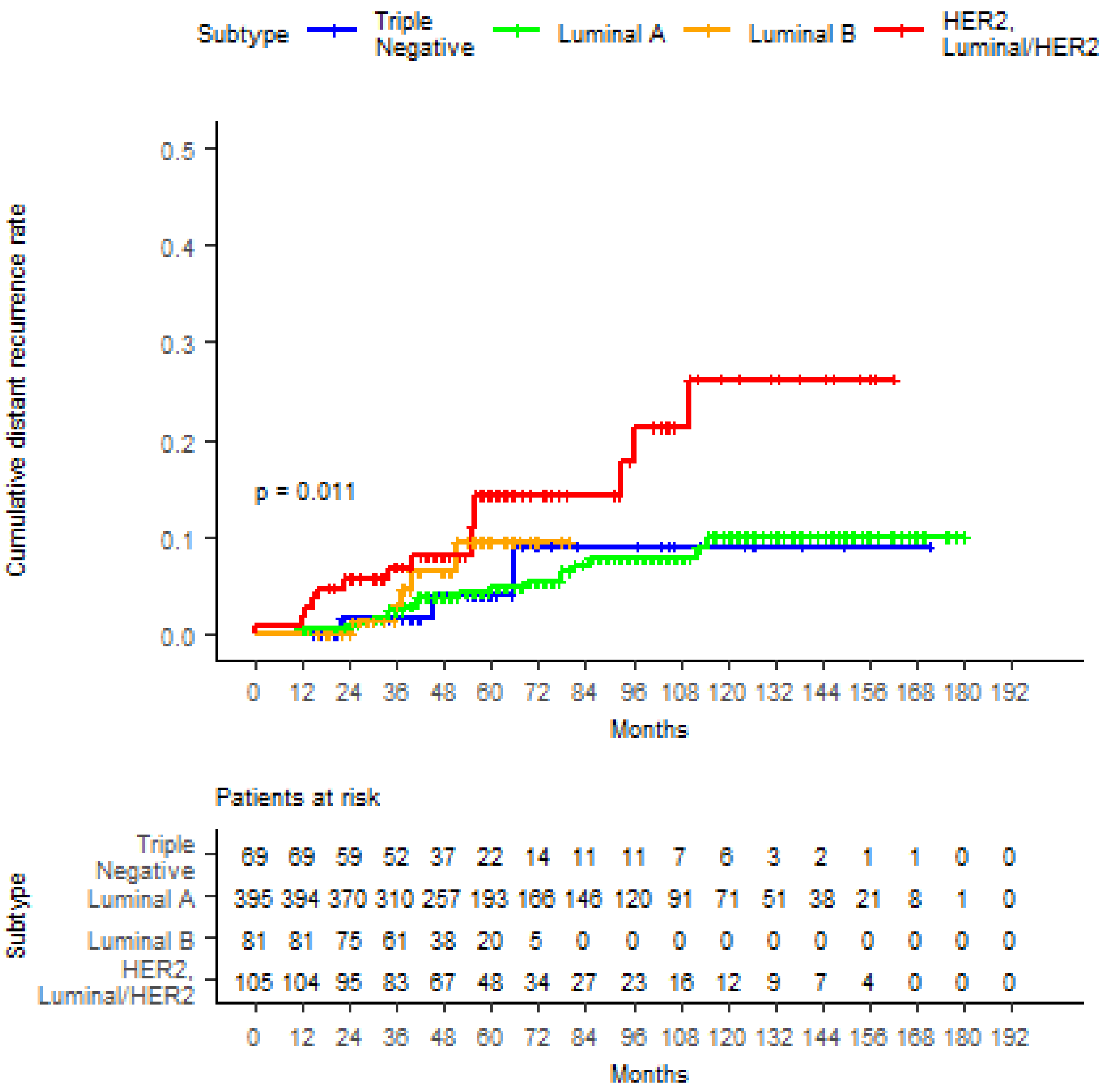

Table 7. Cox regression analysis of characteristics predicting time to distant metastasis.

Variables	HR not adjusted	95% CI	p	HR adjusted*	95% CI	p
**cT**						
cT1	Ref.			Ref.		
cT2	1.38	0.73-2.58	0.319	0.91	0.44-1.86	0.789
cT3	3.79	1.30-11.06	**0.015**	2.85	0.80-10.11	0.106
**cN**						
cN0	Ref.					
cN1	3.29	1.83-5.91	**<0.05**			
**Stage**						
I	Ref.			Ref.		
II	2.66	1.42-4.98	**0.002**	1.60	0.59-4.29	0.348
III	5.88	1.33-25.96	**0.020**	2.23	0.33-15.29	0.415
**Adjuvant chemotherapy**						
No	Ref.			Ref.		
Yes	3.2	1.69-6.08	**<0.05**	1.69	0.79-3.62	0.180
**Fractionation**						
Hypofractionation (>2Gy/Fx)	Ref.					
Normofractionation (1.8-2Gy/Fx)	7.41	1.01-54.31	**0.049**			
**(y)pN**						
(y)pN-	Ref.			Ref.		
(y)pN+	3.39	1.89-6.08	**<0.05**	2.14	0.65-7.04	0.212
**HER2**						
Positive	Ref.			Ref.		
Negative	0.38	0.21-0.72	**0.003**	0.47	0.25-0.89	**0.020**
**Subtype**						
Triple Negative	Ref.					
Luminal A	0.99	0.30-3.35	0.998			
Luminal B	1.77	0.42-7.45	0.435			
HER2,Luminal/HER2	2.83	0.82-9.77	0.101			
